# Clinical practice guideline: sudden hearing loss ‒ The Brazilian taskforce (ABORL/SBO) 2026

**DOI:** 10.1016/j.bjorl.2026.101903

**Published:** 2026-11-05

**Authors:** Fayez Bahmad, Rogério Hamerschmidt, Robinson Koji Tsuji, Arthur Menino Castilho, André Luiz Lopes Sampaio, Denis Rangel, Eduardo Tanaka Massuda, Fabio de Alencar Rodrigues Junior, Felippe Felix, Giulliano Luchi, Gustavo Lara Rezende, Henrique Furlan Pauna, Joel Lavinsky, Juliana Gusmão, Kiara Kalline Medeiros, Lucas Moura Viana, Luiz Rodolpho Penna Lima Júnior, Márcio Salmito, Mariana Leal, Marina Bernardes, Matheus Pedrosa Tavares, Miguel Angelo Hipolyto, Natália Carasek Matos Cascudo, Natália Quinhone Shigematsu, Norma de Oliveira Penido, Pauliana Lamounier, Rafael da Costa Monsanto, Thaís Gomes Abrahão Elias, Vagner Antonio Rodrigues Silva

**Affiliations:** a Associação Brasileira de Otorrinolaringologia e Cirurgia Cérvico-Facial (ABORL-CCF), São Paulo, SP, Brazil; b Sociedade Brasileira de Otologia (SBO), São Paulo, SP, Brazil

**Keywords:** Sudden sensorineural hearing loss, Sudden hearing loss, Clinical practice guideline, Corticosteroids, Intratympanic therapy, Hyperbaric oxygen therapy, Auditory rehabilitation

## Abstract

**Objective:**

To develop evidence-based Brazilian clinical practice recommendations for the evaluation and management of sudden hearing loss, with emphasis on idiopathic sudden sensorineural hearing loss.

**Methods:**

A multidisciplinary Brazilian taskforce composed of otolaryngologists, otologists, and neurotologists was convened by the Brazilian Association of Otorhinolaryngology and Cervico-Facial Surgery and the Brazilian Society of Otology. Each clinical statement was supported by a focused systematic literature review addressing a predefined clinical question. Reviews were conducted according to PRISMA methodology and structured using the PICOS framework. Evidence was synthesized by the panel, and recommendations were formulated considering aggregate evidence quality, benefits and harms, patient preferences, costs, availability of diagnostic and therapeutic resources, and applicability to the Brazilian healthcare context.

**Results:**

Fifteen clinical statements were developed. The guideline recommends early differentiation between conductive and sensorineural hearing loss, recognition of atypical presentations, prompt audiometric confirmation, selective use of computed tomography, magnetic resonance imaging for retrocochlear evaluation, and avoidance of routine laboratory and genetic testing in typical adult-onset idiopathic cases. Systemic corticosteroids, intratympanic corticosteroids, or a combination of both are recommended as initial treatment, while hyperbaric oxygen therapy may be considered in selected patients. Intratympanic corticosteroids are also recommended as salvage therapy. Routine use of antivirals, thrombolytics, vasodilators, vasoactive substances, immunosuppressive agents, immunobiological therapies, neurotrophic factors, stem cell therapies, and other biological agents is not recommended outside specific indications or research settings. Auditory rehabilitation counseling and structured audiometric follow-up are recommended.

**Conclusion:**

This Brazilian guideline provides practical, evidence-based recommendations to standardize the diagnosis, treatment, rehabilitation, and follow-up of patients with sudden hearing loss, aiming to improve timely care and reduce unnecessary interventions.

## Highlights

• Brazilian guideline provides evidence-based recommendations for sudden hearing loss.
• Fifteen clinical statements address diagnosis, treatment, and rehabilitation.
• Audiometry and MRI are emphasized for accurate and timely evaluation.
• Corticosteroids are recommended; selected patients may benefit from HBOT.
• Routine CT, laboratory tests, antivirals, and biologics are discouraged.

## Statement 1 ‒ Exclusion of conductive hearing loss

### Key clinical message

Clinicians should distinguish sensorineural hearing loss from conductive hearing loss at the first presentation of sudden hearing loss.

Initial assessment should include history, otoscopy, bedside tuning fork tests when available, and audiometric confirmation as soon as possible. Excluding conductive causes such as cerumen impaction, otitis media with effusion, hemotympanum, or tympanic membrane perforation is essential to avoid inappropriate treatment and delays in SSNHL management.

## Statement 2 ‒ Differential diagnosis

### Key clinical message

Clinicians should distinguish idiopathic unilateral SSNHL from bilateral, recurrent, and neurologically associated presentations.

Bilateral, recurrent, or neurologically associated sudden hearing loss should be considered clinical red flags. These presentations are more likely to be associated with infectious, autoimmune, vascular, neoplastic, or central nervous system disorders and require individualized diagnostic investigation and, when appropriate, urgent multidisciplinary referral.

## Statement 3 ‒ Imaging in sudden sensorineural hearing loss

### Key clinical message

Clinicians should not routinely obtain computed tomography of the head in the initial evaluation of presumed idiopathic SSNHL.

CT has limited diagnostic yield in uncomplicated idiopathic SSNHL and may expose patients to unnecessary radiation, costs, and incidental findings. CT should be reserved for selected scenarios such as head trauma, suspected temporal bone fracture, intracranial infection, congenital malformations, prior otologic surgery, focal neurological deficits, MRI contraindication, or MRI unavailability.

## Statement 4 ‒ Audiometric confirmation

### Key clinical message

Clinicians should obtain audiometric confirmation of sensorineural hearing loss as soon as possible and within 14-days of symptom onset in adults with suspected sudden hearing loss.

Audiometry confirms the diagnosis, distinguishes sensorineural from conductive or mixed hearing loss, defines severity and configuration, establishes a baseline for prognosis, and guides treatment decisions. Early audiometric confirmation is critical because treatment benefit may be time-dependent.

## Statement 5 ‒ Investigation of retrocochlear pathologies

### Key clinical message

Clinicians should evaluate patients with sudden sensorineural hearing loss for retrocochlear pathology, preferably with magnetic resonance imaging.

MRI of the internal auditory canals and brain is the preferred method to investigate vestibular schwannoma and other retrocochlear disorders. Auditory brainstem response may be considered only when MRI is unavailable or contraindicated, but a negative ABR does not fully exclude retrocochlear disease.

## Statement 6 ‒ Laboratory testing

### Key clinical message

Clinicians should not obtain routine laboratory testing in all patients with SSNHL.

Laboratory testing should be selective and guided by clinical suspicion. Testing may be appropriate in patients with bilateral, recurrent, systemic, autoimmune, infectious, vascular, neurological, or otherwise atypical presentations. Routine indiscriminate testing increases costs and may generate false-positive or clinically irrelevant findings.

## Statement 7 ‒ Genetic evaluation

### Key clinical message

Clinicians should not routinely obtain genetic testing in patients with typical adult-onset idiopathic SSNHL.

Genetic evaluation may be considered in selected patients with bilateral SSNHL, recurrent episodes, family history of hearing loss, pediatric or early-onset presentation, syndromic features, or suspected inherited hearing disorder. Genetic testing should be individualized and ideally accompanied by genetic counseling.

## Statement 8 ‒ Corticosteroid therapy

### Key clinical message

Clinicians should offer corticosteroid therapy as initial treatment for SSNHL.

Systemic corticosteroids, intratympanic corticosteroids, or combined therapy may be considered according to clinical circumstances, severity of hearing loss, contraindications, comorbidities, and patient preferences. Early treatment is emphasized because the therapeutic window is limited.

Sudden sensorineural hearing loss should be approached as a potentially inflammatory and multifactorial inner ear disorder. Corticosteroid therapy should not be understood as a fixed short-course intervention limited to only a few days; instead, treatment duration should be individualized according to disease severity, audiometric response, clinical evolution, comorbidities, and tolerance.

A weight-based regimen, commonly around 1 mg/kg/day of prednisone or equivalent, should also be individualized rather than automatically capped at a universal maximum dose, with careful risk–benefit assessment and appropriate monitoring.

## Statement 9 ‒ Hyperbaric oxygen therapy combined with corticosteroids as initial therapy

### Key clinical message

Clinicians may consider hyperbaric oxygen therapy combined with corticosteroids as initial therapy in selected patients with profound SSNHL.

HBOT may provide benefit through increased cochlear oxygen delivery, particularly when initiated early in patients with severe or profound loss. Its use should be individualized because of low-certainty evidence, high cost, limited availability, treatment burden, and potential adverse events such as middle ear barotrauma, sinus barotrauma, claustrophobia, and transient visual symptoms.

## Statement 10 ‒ Intratympanic corticosteroid salvage therapy

### Key clinical message

Clinicians should offer, or refer to a clinician who can offer, intratympanic corticosteroid therapy to patients with incomplete recovery from SSNHL.

Intratympanic therapy is recommended as salvage treatment after incomplete response to initial management. It may improve hearing outcomes while limiting systemic steroid exposure and should be discussed with patients in the context of expected benefit, procedural discomfort, tympanic membrane risks, and local expertise.

## Statement 11 ‒ Hyperbaric oxygen therapy as rescue therapy

### Key clinical message

Clinicians may offer hyperbaric oxygen therapy, in combination with corticosteroids, as salvage therapy for patients with SSNHL and incomplete recovery after initial treatment.

HBOT as rescue therapy should preferably be considered within the early post-onset period and mainly in selected patients with severe or profound residual hearing loss. Decisions should account for local availability, patient tolerance, cost, contraindications, and the limited certainty of current evidence.

## Statement 12 ‒ Antivirals, thrombolytics, vasodilators, and vasoactive substances

### Key clinical message

Clinicians should not routinely prescribe antivirals, thrombolytics, vasodilators, vasoactive substances, or related agents for idiopathic SSNHL.

Current evidence does not consistently demonstrate meaningful hearing benefit from these therapies in routine idiopathic cases. Their use may expose patients to adverse events, bleeding risk, cardiovascular effects, drug interactions, and unnecessary costs. Targeted treatment may be appropriate only when a specific underlying diagnosis independently justifies it.

## Statement 13 ‒ Other pharmacological therapies: immunosuppressors and immunobiological agents

### Key clinical message

Clinicians should not routinely prescribe immunosuppressive agents, immunobiological therapies, neurotrophic factors, stem cell therapies, or other biological agents for SSNHL outside approved indications or research protocols.

Although these therapies are biologically plausible and may be relevant to future precision medicine strategies, current clinical evidence is insufficient to support routine use in idiopathic SSNHL. Their use should be restricted to selected etiologies, specialist evaluation, or controlled clinical research.

## Statement 14 ‒ Auditory rehabilitation

### Key Clinical Message

Clinicians should counsel patients with persistent hearing loss and/or tinnitus after SSNHL regarding auditory rehabilitation options.

Rehabilitation should be individualized according to residual hearing, speech discrimination, tinnitus burden, communication needs, and patient preference. Options may include hearing aids, CROS systems, bone conduction devices, cochlear implantation, tinnitus counseling, auditory training, and communication strategies.

## Statement 15 ‒ Routine post-treatment follow-up

### Key clinical message

Clinicians should obtain follow-up audiometric evaluation after treatment completion, within 2- to 3-months, and within 6-months after SSNHL onset.

Follow-up audiometry documents hearing recovery, identifies delayed improvement or deterioration, guides salvage therapy and rehabilitation, and supports long-term counseling. Additional follow-up should be individualized for patients with persistent hearing loss, tinnitus, vestibular symptoms, recurrent episodes, or suspected secondary etiologies.

## Introduction

Sudden Sensorineural Hearing Loss (SSNHL) is an otologic emergency defined by a rapid onset of hearing loss.^1^ It is characterized by hearing loss greater than 30 dB across at least three contiguous frequencies occurring within a 72-h window.^2^ It occurs mostly unilaterally and its incidence varies by region, reaching up to 60 per 100,000 inhabitants/year. The prevalence among patients older than 65-years may reach 77 per 100,000.^3^^,^^4^ There are still no consolidated data for the Brazilian population, reinforcing the importance of national registries.

Despite its relatively high incidence, the underlying etiology remains idiopathic in the majority of cases, with proposed mechanisms including viral infections, microvascular compromise, autoimmune inner ear disease, and cochlear synaptopathy.^5^, ^6^, ^7^, ^8^ Current first-line treatment relies on corticosteroids, administered systemically or intratympanically, based on their broad anti-inflammatory and immunosuppressive properties.^1^ However, up to 30%–50% of patients fail to achieve complete recovery, highlighting a need for alternative or adjunctive therapeutic approaches.^2^

This guideline, developed by the Brazilian Association of Otorhinolaryngology and Head and Neck Surgery and the Brazilian Society of Otology, aims to provide evidence-based recommendations for the evaluation of patients with sudden sensorineural hearing loss, with particular emphasis on the management of idiopathic sudden sensorineural hearing loss.

The most recent update to the guidelines was published in 2019 in Otolaryngology-Head and Neck Surgery Volume 161, Issue 1_suppl, August 2019, Pages S1-S45, which updated the then-current Clinical Practice Guideline Development Manual, Third Edition, of the American Academy of Otolaryngology-Head and Neck Surgery Foundation (Rosenfeld et al. Otolaryngol Head Neck Surg. 2013;148^1^:S1-S55).

ABORL-CCF and SBO understand that a new update is necessary, this time to be carried out by Brazilian specialists. Focusing on opportunities for quality improvement, this guideline should incorporate new forms of treatment, such as immunobiologicals described in recent years, as well as improve diagnostic accuracy, facilitate rapid intervention by specialists, reduce variations in treatment to improve hearing and rehabilitation outcomes for affected patients, and reduce unnecessary examinations and imaging procedures.

## Methods

Following best practices for developing clinical practice guidelines, all topics within the statements of practical actions were subjected to a rigorous systematic literature review to enable the production of this clinical practice guideline on sudden hearing loss. For this, we gathered representatives from the areas of general otolaryngology, otology, and neurotology to act as co-authors, conducting Systematic Reviews on the main topics to be updated and defined by the guideline.

All systematic reviews were conceived according to the Cochrane Handbook for Systematic Reviews of Interventions^1^ and conducted following the Preferred Reporting Items for Systematic Reviews and Meta-Analyses (PRISMA) methodology.^2^ The eligibility criteria included studies through a PICOS strategy (an acronym in which P denotes population; I, Intervention (or exposure); C, Comparison; O, Outcome; and S, Study type).^2^ The population of interest was “SSNHL patients”, the intervention, comparison, and outcome varied between each research question. The study designs included randomized clinical trials, quasi experimental studies, cohort studies, and systematic reviews with or without meta-analysis.

Studies including patients younger than 18-years or using a definition of SSNHL different from the prespecified criteria were excluded: “Sensorineural hearing loss that occurs within a 72-h window and meets audiometric criteria of a decrease in hearing of 30-decibels affecting at least 3-consecutive frequencies. If pre-deafness audiometry is unavailable, hearing loss can be defined in relation to the thresholds of the opposite ear”.

The search strategy used Medical Subject Headings (MeSH) vocabulary in English and its alternative descriptors. Pilot trials were done in the PubMed database to assess the feasibility of the proposal. The ultimate search swept electronic databases: PubMed (MEDLINE), SciELO, Lilacs, Embase, Web of Science and Cochrane Library. The unique references remaining went through a selection process by two blinded reviewers and risk of bias was assessed. The methodological design in this study used secondary data analysis and was conducted by using a thorough process, reinforcing research ethics in all of its stages.

After all systematic reviews were completed, all data were compiled to formulate the statements, based on recent literature and the highest level of evidence studies. Each clinical statement was supported by a focused systematic review addressing a predefined clinical question. Search strategies, eligibility criteria, number of records identified, and included studies varied according to the topic. The panel synthesized the evidence and assigned the aggregate evidence quality and policy level for each recommendation.

## Statement 1 ‒ Exclusion of Conductive Hearing Loss (CHL)

Clinicians should distinguish SSNHL from CHL when a patient first presents with SHL. Strong recommendation.

### Action statement

Quality improvement opportunity: Improve diagnostic accuracy at the initial presentation of sudden hearing loss, ensuring prompt identification of patients with SSNHL who require urgent evaluation and treatment while avoiding inappropriate management of patients with conductive hearing loss.

Aggregate evidence quality: Grade B, based on observational studies, diagnostic accuracy studies, established clinical practice guidelines, and consistent evidence supporting the ability of history, otoscopy, tuning fork tests, and audiometry to distinguish conductive from sensorineural hearing loss.

Level of confidence in the evidence: High.

Benefits:

• Improve diagnostic accuracy.
• Facilitate timely diagnosis of SSNHL.
• Avoid delays in treatment initiation.
• Identify conductive causes of hearing loss that may be readily treatable.
• Improve patient counseling and prognostic assessment.
• Reduce inappropriate diagnostic testing and therapeutic interventions.
• Facilitate appropriate referral to otolaryngology specialists.

Risks, harms, costs: Minimal. Potential risks include misclassification when relying solely on bedside examination without audiometric confirmation, and costs associated with audiologic assessment. These risks are outweighed by the benefits of accurate diagnosis.

Benefits-harm assessment: Preponderance of benefit over harm.

Value judgments: The guideline panel placed a high value on early differentiation between conductive and sensorineural hearing loss because treatment pathways, urgency of intervention, prognosis, and need for additional investigations differ substantially. Although high-quality randomized studies are lacking, this distinction is considered a fundamental prerequisite for appropriate management of sudden hearing loss.

Intentional vagueness: The specific diagnostic tools used to distinguish CHL from SSNHL may vary according to local resources and clinical setting. While audiometry remains the reference standard, clinicians may use otoscopy, tuning fork tests, tympanometry, smartphone-based tools, or other validated methods during the initial evaluation.

Role of patient preferences: Limited. Patients may participate in decisions regarding the timing and setting of audiologic evaluation; however, differentiation between conductive and sensorineural hearing loss is considered an essential component of appropriate clinical care.

Exceptions: None.

Policy level: Strong recommendation.

Differences of opinion: None.

### Supporting text

This statement emphasizes that differentiating between CHL and SSNHL is crucial for guiding treatment options and prognosis because management strategies for these conditions differ substantially. As published in prior guidelines, there is strong evidence that CHL causes such as cerumen impaction or middle ear effusion can be easily treated and has excellent prognosis.^1^^,^^2^

Sudden hearing loss is sensorineural in nature. This guideline addresses diagnostic and therapeutic considerations exclusively for this condition. The exclusion of conductive causes is therefore essential to ensure accurate evaluation and appropriate clinical management.

Most conductive issues are more readily managed. The most evident causes of sudden conductive hearing loss, such as cerumen impaction, acute otitis media, otitis media with effusion, hemotympanum, and traumatic tympanic membrane perforations, can be readily excluded through a careful history and otoscopic examination. Clinicians must perform physical examination ‒ including tuning fork tests ‒ and, ultimately, audiometry, which may not be readily available at the initial stage of investigation. Pneumatic otoscopy and tympanometry can also help guide diagnosis.^2^

Current guidelines in patients with sudden hearing loss, clinicians should obtain, or refer to a clinician who can obtain, audiometry as soon as possible ‒ within 14-days of symptom onset ‒ to exclude conductive hearing loss and confirm the diagnosis of sudden sensorineural hearing loss.^3^ However, it is known that, in clinical practice, the audiometry may not be a routine part of the patient’s initial assessment.

A study published in 2025 analyzed adherence to the AAO-HNSF Clinical Practice Guidelines for sudden hearing loss. Fewer than two-thirds of the 24203 patients evaluated underwent audiometry, and only 35.8% received it within one month of symptom onset.^4^

Anamnesis: The initial evaluation of patients with sudden sensorineural hearing loss should focus on excluding conductive causes of hearing impairment. This assessment includes obtaining a detailed medical history. Patients lack the capacity to accurately categorize their hearing impairment as originating from conductive or sensorineural pathways through self-assessment. Tinnitus and/or vertigo may occur. A thorough clinical interview should include inquiries regarding antecedent head or ear trauma, auricular discomfort, ear canal instrumentation, purulent discharge from the ear, fever, and any neurological deficits or systemic symptoms.

Otoscopy: While certain etiologies of conductive hearing loss may present with normal otoscopic findings, sudden-onset conductive hearing loss is almost invariably associated with identifiable otoscopic abnormalities, including cerumen impaction and otitis media with effusion.

In many cases, the initial assessment of patients with sudden hearing loss is conducted by non-specialists. Performing a comprehensive otoscopic examination in such contexts can be challenging, as it requires specific expertise to accurately evaluate the external auditory canal and tympanic membrane. General practitioners correctly diagnosed the pathology in only 41% of ears, compared with otolaryngologists, who served as the reference standard.^5^

These limitations may hinder the timely identification of conditions that mimic or contribute to sudden hearing loss. In contexts where access to specialists is limited, artificial intelligence-based tools may assist clinicians in providing more accurate and prompt diagnoses. By replicating aspects of human cognition ‒ particularly the ability to recognize patterns and detect abnormalities ‒ artificial intelligence holds promise as a valuable adjunct in the diagnostic process.^6^

We conducted a systematic review on artificial intelligence in otoscopic diagnosis. The databases used included Medline via PubMed, Latin American and Caribbean Health Sciences Literature (LILACS) via the Virtual Health Library (VHL), Scopus, Embase, Web of Science, and the grey literature of Google Scholar and ProQuest Dissertations & Theses. The search strategies were composed of Medical Subject Headings (MeSH), Health Sciences Descriptors (DeCS), as well as free terms, properly connected by the Boolean operators AND and OR. These terms were adapted for each database using the following keywords in the search strategy: "otoscopy", "otoscopic examination", "otoscopic image", "video-otoscopy", "videootoscopy", "digital otoscopy", "telemedicine", "telehealth", "remote consultation", "teleconsultation", "mobile health", "mobile phone", "smartphone", "artificial intelligence", "AI", "machine learning", and "deep learning". Filtering was also based on publication date (limiting to studies published in the last 10-years).

This systematic review identified 37 studies investigating the application of Artificial Intelligence (AI) algorithms for the diagnosis of otologic conditions from otoscopic images and videos. The studies analyzed a total of over 120,000 images, employing various deep learning architectures, predominantly Convolutional Neural Networks (CNNs), to automate the detection and classification of middle and external ear pathologies, with most studies focusing on pediatric populations, particularly for the diagnosis of otitis media and its variants.

The diagnostic accuracy of AI algorithms demonstrated considerable variation across studies, ranging from 73% to 99.54%, with a notable trend toward improved performance in more recent publications. Early studies, such as Habib et al. (2020)^7^ and Tsutsumi et al. (2021),^8^ reported moderate accuracies of 76% and 73 %–77 %, respectively. However, subsequent investigations showed marked improvements, with several studies from 2024 to 2025 achieving accuracies exceeding 98%, including Azam et al. (2024) at 99.38%, Cömert et al. (2024) at 99.37%, Rao et al. (2024) at 99.3%, and Elmorsy et al. (2025) at 99.54%.^9^, ^10^, ^11^, ^12^ Sensitivity values ranged from 58% to 99%, while specificity ranged from 58% to 99.36%, indicating robust discriminatory capacity in most algorithms.

Notably, several studies specifically reported superior AI performance compared to human clinicians. Cha et al. demonstrated AI accuracy of 77.14% compared to 71.17% for ENT specialists and 45.63% for non-ENT physicians.^13^ Wang et al. reported AI accuracy of 81.7% versus 67.8% for clinicians, and Suresh et al. demonstrated the most striking difference, with AI achieving 90.6% accuracy compared to 59.4% for pediatricians using standard handheld otoscopes.^14^^,^^15^

Despite that, the otolaryngologist's assessment remains superior. In the study by Noda, despite the AI system (GPT-4 V) achieving 82.1% accuracy in diagnosing middle ear diseases, otolaryngologists demonstrated superior performance with accuracy rates exceeding 95%, reinforcing that specialist clinical assessment remains the gold standard for otoscopic diagnosis.^20^

Based on this systematic review data, artificial intelligence tools demonstrate significant potential to help exclude conductive causes of hearing loss in the context of sudden sensorineural hearing loss, especially in primary care. The AI-based diagnostic systems analyzed in this review show high accuracy rates in detecting various ear pathologies. These automated otoscopic assessment tools can rapidly identify conductive abnormalities that might otherwise mimic sudden hearing loss presentations, enabling clinicians to quickly rule out treatable middle ear conditions and expedite appropriate management for true sensorineural emergencies.^13^, ^14^, ^15^, ^16^ The development of smartphone-based and web-accessible diagnostic tools represents a paradigm shift toward democratized healthcare, potentially enabling earlier diagnosis and intervention of sudden hearing loss.^8^^,^^17^, ^18^, ^19^

Tuning fork tests: The tuning fork tests (Weber and Rinne) play a crucial role in the initial assessment of sudden hearing loss by helping to differentiate between conductive and sensorineural causes at the bedside.

This clinical examination tool is particularly valuable in urgent scenarios as it can quickly exclude conductive causes such as middle ear effusion thereby supporting the diagnosis of SSNHL and facilitating prompt corticosteroid treatment and specialist referral.

Abdullah, in 2022, compared the diagnostic accuracy of Audiometric Weber (AW) and Tuning Fork Weber (TFW) tests against pure-tone audiometry in 74 patients with conductive hearing loss. Both tests demonstrated substantial agreement with audiometry (kappa 0.63‒0.72) and high accuracy rates (81 %–87 %).^21^

Despite that, in a study analyzing diagnostic and management practices for SSNHL among Canadian family physicians, tuning fork tests were employed by only 61.5% of respondents to inform clinical decisions, demonstrating marked under-utilization.^22^ The under-utilization of tuning fork tests in primary care can be partially attributed to insufficient availability of this diagnostic equipment and to the lack of training. To address this limitation, a prospective study evaluated a Smartphone-based Weber Test (SPWT) as an alternative to the traditional tuning fork Weber test, enrolling 60 patients with unilateral hearing loss in the emergency department. The SPWT, which uses the smartphone's vibration application pressed against the forehead, demonstrated agreement with the traditional tuning fork test in 92% of cases, with sensitivity and specificity of 76.9% and 97.1%, respectively.^23^ Hopkins, in 2019, also evaluated whether smartphone vibration applications could serve as a valid alternative to tuning forks for Weber's test in 20 healthy participants using earplugs to simulate conductive hearing loss. The iPhone application (iBrateMe) demonstrated equivalent accuracy to the 512 Hz tuning fork, with no statistically significant differences in sound lateralization for either ear occlusion (p = 0.147 and p > 0.999). This could be a viable and more accessible alternative for assessing conductive hearing loss.^24^

Another alternative to the Weber tuning fork test is the hum test, in which the patient is asked to hum and if he or she hears one’s own hum louder in the affected ear, it is likely CHL in that ear. Ahmed compared the Hum Test against the Weber Test in 29 participants with simulated conductive hearing loss. The Hum Test showed sensitivity of 89.7 %–93.1 % and specificity of 100%, comparable to the Weber Test (96.6% sensitivity, 100% specificity).^25^ Neither of these has the accuracy of audiometry, but each can be used in the initial assessment if better testing methods are not available.

Statement 2 ‒ Differential diagnosis

Clinicians should distinguish idiopathic unilateral, bilateral, recurrent, and neurologically associated presentations. Strong recommendation.

### Action statement

Quality improvement opportunity: Improve recognition of atypical presentations of SSNHL that are associated with secondary etiologies, poorer prognosis, and the need for urgent diagnostic investigation and multidisciplinary management.

Aggregate evidence quality: Grade B, based on observational studies, systematic reviews, clinical practice guidelines, and consistent evidence demonstrating that bilateral, recurrent, and neurologically associated SSNHL are more frequently linked to identifiable underlying diseases than idiopathic unilateral SSNHL.

Level of confidence in the evidence: High.

Benefits: Early recognition of atypical presentations of SSNHL may facilitate identification of secondary etiologies, improve diagnostic accuracy and risk stratification, and promote timely investigation of infectious, autoimmune, vascular, neoplastic, and neurological disorders. Appropriate evaluation may reduce delays in the diagnosis of potentially serious conditions, including stroke and central nervous system diseases, improve patient counseling regarding prognosis and expected outcomes, facilitate referral to appropriate specialists when indicated, and support individualized diagnostic and therapeutic decision-making.

Risks, harms, costs: Recognition of atypical presentations may lead to increased use of laboratory investigations, imaging studies, and specialist consultations, with a consequent increase in healthcare costs. Additional diagnostic testing may also result in incidental findings that require further evaluation. However, these potential harms are generally outweighed by the benefits of identifying clinically significant underlying disorders that may require specific treatment or urgent intervention.

Benefits-harm assessment: Preponderance of benefit over harm.

Value judgments: The guideline panel placed a high value on recognizing atypical SSNHL presentations as clinical red flags. Although idiopathic unilateral SSNHL remains the most common presentation, bilateral, recurrent, and neurologically associated cases are disproportionately associated with systemic, autoimmune, infectious, vascular, and neoplastic diseases that require specific management beyond standard corticosteroid therapy.

Intentional vagueness: The extent of the additional diagnostic evaluation should be individualized according to the patient's clinical presentation, associated symptoms, risk factors, and local resource availability. No specific diagnostic algorithm is mandated for all atypical presentations.

Role of patient preferences: Limited. Patients may participate in decisions regarding the extent of diagnostic testing and specialist referrals; however, recognition of atypical presentations and appropriate investigation are considered essential components of evidence-based care.

Exceptions: None.

Policy level: Strong recommendation.

Differences of opinion: None.

**Supporting text**

## Introduction

Sudden Sensorineural Hearing Loss (SSNHL) is defined as a rapid-onset sensorineural hearing loss of at least 30 dB across three consecutive frequencies within 72 -hs.^26^, ^27^, ^28^ While idiopathic unilateral SSNHL remains the focus of current clinical guidelines,^3^ atypical presentations including bilateral, recurrent, and neurologically associated forms warrant particular attention due to their prognostic implications and association with secondary causes.^29^ The purpose of this review is to compare idiopathic unilateral SSNHL with these atypical variants, highlighting differential diagnoses, clinical reasoning, grade of evidence, and management strategies.

## Methods

This systematic review was registered in the International Prospective Register of Systematic Reviews (PROSPERO; CRD420251086642). Informed consent was waived because the data were available and legally accessible to the public and appropriately protected by law. This study followed the Preferred Reporting Items for Systematic Reviews and Meta-Analyses (PRISMA) reporting guideline.

### Search strategy

PubMed/MEDLINE, clinical trial registry (https://clinicaltrials.gov/), Embase, Scopus, and Web of Science databases were searched using MeSH terms: ("Hearing Loss, Sudden"[Mesh] OR "sudden sensorineural hearing loss" OR "sudden hearing loss" OR "acute sensorineural hearing loss") AND (viral OR "herpes simplex virus" OR "varicella zoster virus" OR HIV OR "human immunodeficiency virus" OR bacterial OR mycoplasma OR lyme OR "Lyme disease"[Mesh] OR tuberculosis OR syphilis OR fungal OR "autoimmune inner ear disease" OR autoimmune OR ototoxic OR trauma OR "lead poisoning" OR genetic OR "mitochondrial disorders" OR MELAS OR stroke OR "Cogan Syndrome"[Mesh] OR "neurofibromatosis 2" OR vestibular schwannoma OR "carcinomatous meningitis" OR "lymphomatosis" OR sarcoidosis OR hyperviscosity OR "Ménière Disease"[Mesh] OR "multiple sclerosis"[Mesh]). The reference lists of selected articles were also searched for additional articles.

### Study selection

We included observational studies and prospective randomized controlled trials involving adult (over 18-years-old) participants, published in English from January 2015 until July 2025, and using the diagnostic criteria outlined by the American Academy of Otolaryngology-Head and Neck Surgery. The search was restricted to studies comparing differential diagnosis among idiopathic unilateral SSNHL, bilateral hearing loss, fluctuating hearing loss, and sudden deafness with neurological findings.

### Data extraction

Two investigators independently screened the articles and extracted data from the included studies using a standard data extraction form. For each study, the year of publication; authors; study design; sample sizes; participants’ age and sex distribution; severity of hearing loss; and differential features among idiopathic unilateral SSNHL, bilateral hearing loss, fluctuating hearing loss, and sudden deafness with neurological findings were recorded.

### Grade of evidence definitions

Two researchers evaluated the grade of evidence in the included studies. The assessment covers five domains: (A) Strong recommendation, high-quality evidence; (B) Moderate recommendation, moderate-quality evidence; (C) Weak recommendation, low-quality or inconsistent evidence; (D) Recommendation against use; (I) Insufficient evidence to recommend for or against use.

## Results

### Search yield

Our initial search of scientific databases yielded 2325 records. A total of 1611 were removed as duplicates. After screening 575 articles, 545 were excluded for not meeting the inclusion criteria. Ultimately, 30 articles were included in this review.

Idiopathic unilateral SSNHL

Idiopathic unilateral SSNHL represents 70%–90% of cases depending on the series.^28^^,^^30^ Patients typically present with sudden unilateral hearing loss, often accompanied by tinnitus and vertigo. Approximately one-third recover spontaneously,^30^^,^^31^ though recovery is influenced by age, degree of hearing loss, and presence of vertigo. Guideline-based management includes early audiometric confirmation, systemic and/or intratympanic steroids, and Magnetic Resonance Imaging (MRI) to exclude retrocochlear pathology.^3^^,^^32^ Prognosis is generally favorable compared with atypical forms.

History should address onset (abrupt vs. progressive), laterality (unilateral vs bilateral), associated symptoms (tinnitus, vertigo, aural fullness), and relevant exposures (ototoxic drugs, recent infections, trauma).^3^^,^^26^, ^27^, ^28^^,^^30^, ^31^, ^32^ Physical examination is often normal, though bedside tuning fork tests (Rinne and Weber) help confirm a sensorineural origin.^3^^,^^32^

### Grade of evidence: B

Bilateral SSNHL

Bilateral SSNHL is rare and typically non-idiopathic.^33^^,^^34^ Reported causes include viral infections (Coronavirus Disease ‒ COVID-19, herpes viruses, HIV, syphilis, Lyme disease), autoimmune inner ear disease, systemic inflammatory conditions (Cogan’s syndrome, Susac Syndrome, sarcoidosis), ototoxic medications, trauma, and metabolic or mitochondrial disorders. Bilateral SSNHL may also herald vascular events, including anterior inferior cerebellar artery infarction.^35^ Prognosis is often poorer than in unilateral idiopathic cases, and systemic evaluation is mandatory.

A clinician should be alert to systemic symptoms such as fever, rash, arthralgias, neurological complaints, or recent infections.^33^^,^^34^^,^^36^^,^^37^ Medication history (aminoglycosides, cisplatin, loop diuretics) is critical.^38^^,^^39^ On examination, bilateral reduction of hearing confirmed by audiometry suggests non-idiopathic etiologies.^33^^,^^34^ Signs of systemic disease (skin lesions, lymphadenopathy, neurologic deficits) help guide further testing.^40^^,^^41^

### Grade of evidence: C

Recurrent SSNHL

Recurrent episodes of SSNHL suggest underlying pathology.^42^^,^^43^ Common causes include Ménière’s disease, autoimmune inner ear disease, vestibular schwannoma, and vascular disorders. The probability of hearing recovery diminishes with subsequent episodes, with reported recovery rates falling below 10% after the third episode in vestibular schwannoma–associated SSNHL.^44^ Long-term follow-up and repeated audiometric evaluation are crucial in this group.

In recurrent cases, physicians should ask about episodic vertigo, fluctuating tinnitus, or aural fullness (suggesting Ménière’s disease)^42^; personal or family history of autoimmune disease;^45^ and progressive unilateral hearing loss (raising suspicion for vestibular schwannoma).^44^^,^^46^^,^^47^ Physical examination may reveal imbalance or abnormal vestibular testing.^48^ Repeated audiometry helps document fluctuating patterns.^42^^,^^45^

### Grade of evidence: C

SSNHL with neurological findings

SSNHL accompanied by focal neurological signs strongly indicates central nervous system involvement.^49^^,^^50^ Posterior circulation strokes, especially anterior inferior cerebellar artery infarctions, may present initially with SSNHL and vertigo.^35^^,^^43^^,^^51^ Clinicians should recognize that sudden sensorineural hearing loss may be the earliest manifestation of Anterior Inferior Cerebellar Artery (AICA) infarction, preceding overt neurological deficits by hours or even days.

Other causes include multiple sclerosis, central nervous system infections (bacterial meningitis, carcinomatous meningitis), and tumors of the cerebellopontine angle such as vestibular schwannomas and meningiomas.^50^^,^^52^, ^53^, ^54^ These cases demand urgent neuroimaging and neurologic evaluation, as delayed recognition may lead to irreversible deficits.

The clinician should assess for acute vertigo, diplopia, facial weakness, dysarthria, gait instability, or sensory changes.^35^^,^^43^^,^^51^ History of vascular risk factors (hypertension, diabetes, atrial fibrillation, smoking) increases suspicion of stroke.^43^ Neurological examination may reveal cranial nerve deficits, cerebellar signs, or long tract involvement, which mandate urgent neuroimaging.^50^^,^^52^, ^53^, ^54^

## Grade of evidence: B

### Discussion

Idiopathic unilateral SSNHL, though distressing, follows a relatively standardized diagnostic and therapeutic pathway as outlined in the AAO-HNSF guideline.^3^ In contrast, bilateral, recurrent, and neurologically associated SSNHL serve as clinical “red flags” that often signal secondary etiologies. These atypical forms require a broadened diagnostic workup, including serology, autoimmune panels, vascular imaging, and cerebrospinal fluid analysis when indicated.^31^^,^^34^

Prognosis is generally less favorable compared to idiopathic unilateral SSNHL, particularly in recurrent and bilateral cases.^44^ Recognition of these variants is critical for timely intervention. For example, bilateral SSNHL may prompt investigation for systemic autoimmune disease, while SSNHL with neurological findings should raise suspicion for stroke or demyelinating disease.^35^^,^^43^

### Conclusion

Idiopathic unilateral SSNHL remains the most common and best-studied presentation, with guideline-based therapies focusing on corticosteroid administration and exclusion of retrocochlear pathology. In contrast, bilateral, recurrent, and neurologically associated SSNHL are uncommon but clinically significant, often indicating underlying systemic, infectious, autoimmune, neoplastic, or vascular disease.

These forms mandate comprehensive evaluation and carry a poorer prognosis. Patients with atypical forms (bilateral, recurrent, neurological) should be considered urgent referrals and undergo extended workup.

Future research should focus on developing biomarkers and imaging strategies to distinguish idiopathic from secondary SSNHL at presentation, thereby improving patient outcomes.

## Statement 3 ‒ Imaging in sudden sensorineural hearing loss

Clinicians should not routinely obtain computed tomography of the head in the initial evaluation of patients with presumed idiopathic sudden sensorineural hearing loss.

### Action statement

Quality improvement opportunity: Reduce unnecessary use of computed tomography in the initial evaluation of presumed idiopathic SSNHL, promote evidence-based imaging practices, minimize radiation exposure, optimize healthcare resource utilization, and facilitate appropriate selection of patients who may benefit from imaging.

Aggregate evidence quality: Grade B, based on clinical practice guidelines, systematic reviews, observational studies, and consistent evidence demonstrating low diagnostic yield and limited impact of routine computed tomography on clinical management in patients with presumed idiopathic SSNHL.

Level of confidence in the evidence: High.

Benefits: Reduce unnecessary imaging studies; decrease patient exposure to ionizing radiation; reduce healthcare costs; avoid incidental findings leading to unnecessary investigations; encourage appropriate utilization of magnetic resonance imaging when retrocochlear pathology is suspected; improve adherence to evidence-based diagnostic pathways; facilitate selective identification of patients with trauma, infection, structural abnormalities, or neurological findings who may benefit from CT imaging.

Risks, harms, costs: Potential failure to identify uncommon structural abnormalities when CT is not performed in selected patients; possible delay in diagnosis in situations where MRI is unavailable; costs associated with alternative imaging modalities. These risks are minimized through appropriate patient selection and clinical judgment.

Benefits-harm assessment: Preponderance of benefit over harm.

Value judgments: The guideline panel placed a high value on avoiding unnecessary imaging studies that do not alter clinical management while recognizing that CT remains useful in selected clinical scenarios, including head trauma, suspected temporal bone pathology, intracranial infection, acute neurologic deficits, congenital malformations, and situations in which MRI is unavailable or contraindicated.

Intentional vagueness: The statement does not define specific clinical thresholds for obtaining CT imaging in every circumstance. The decision should be individualized based on clinical presentation, associated symptoms, patient age, local resource availability, and physician judgment.

Role of patient preferences: Limited. Patients may participate in discussions regarding imaging options, radiation exposure, costs, and diagnostic expectations; however, the decision to perform CT should primarily be guided by clinical indications and evidence-based recommendations.

Exceptions: Patients with head trauma, suspected temporal bone fracture, intracranial infection, acute focal neurological deficits, congenital ear malformations, prior otologic surgery, contraindications to MRI, or situations in which MRI is unavailable.

Policy level: Recommendation against routine use of computed tomography in the initial evaluation of presumed idiopathic SSNHL; recommendation for selective use in specific clinical scenarios. CT should not delay corticosteroid treatment.

Differences of opinion: None.

**Supporting text**

## Introduction

Although most cases are idiopathic, retrocochlear, inflammatory, infectious, vascular, and structural etiologies must be considered during the diagnostic work-up. Early etiologic investigation is therefore essential to optimize auditory recovery and long-term functional outcomes.

International clinical practice guidelines recommend Magnetic Resonance Imaging (MRI) as the preferred modality for retrocochlear assessment and advise against routine Computed Tomography (CT) in the initial evaluation of presumed idiopathic SSNHL.^3^ This recommendation is based on the limited diagnostic yield of CT and its negligible impact on therapeutic decision-making.^55^ Despite this, CT remains frequently used in emergency and vestibular presentations, highlighting a persistent gap between guideline-directed care and real-world clinical practice.^56^^,^^61^

## Methods – PRISMA-Based systematic review design

This consensus section is grounded in a systematic review conducted according to PRISMA-P principles, specifically designed to evaluate the diagnostic and clinical utility of CT in SSNHL.

### Data sources and search strategy

Literature searches were performed across PubMed/MEDLINE, Scopus, Cochrane Library, and Google Scholar, targeting publications from 2019 to 2024 in English or Portuguese.

### Eligibility criteria (PICO framework)

Population ‒ patients with SSNHL of any age; Intervention ‒ CT as initial imaging; Comparison ‒ MRI or no imaging; Outcomes ‒ diagnostic yield, detection of structural abnormalities, and clinical impact on management. Eligible designs included clinical guidelines, systematic reviews, observational studies, and cohort analyses.

### Study selection (PRISMA)

110 records were identified; 34 remained after duplicate removal and screening; 14 full-text articles were assessed; 10 studies were included. Because two studies corresponded to the same publication, the qualitative synthesis comprised 9 unique studies.

### Evidence synthesis

#### Adult population

Observational evidence indicates that a substantial proportion of adults with SSNHL undergo CT imaging, particularly in emergency settings and in the presence of vestibular symptoms.^56^^,^^61^ Diagnostic yield is low and CT rarely changes management in presumed idiopathic SSNHL, supporting restrictive, indication-based use.^3^^,^^55^

#### Pediatric population

Imaging considerations differ in children due to the higher prevalence of congenital and structural abnormalities. Imaging may detect abnormalities in approximately one-third of pediatric cases; however, therapeutic consequences are uncommon and benefits are largely limited to prognostic and hereditary counseling.^58^^,^^60^ Selective imaging is therefore favored over routine CT.

#### Safety

Ionizing radiation exposure from CT during childhood is associated with increased long-term cancer risk, reinforcing that CT should be restricted to clearly justified clinical scenarios and performed using the lowest achievable radiation dose.^55^^,^^58^

#### Clinical interpretation

Across guideline statements, systematic reviews, and observational studies, the evidence consistently demonstrates low diagnostic yield of routine CT in adult idiopathic SSNHL; moderate structural detection in pediatric patients with limited therapeutic impact; meaningful radiation-related risk in children; and clear superiority of MRI for retrocochlear evaluation.^3^^,^^55^, ^56^, ^57^, ^58^, ^59^, ^60^

Collectively, these findings support a restrictive, indication-based role for CT in SSNHL.

#### Research gaps and future directions

Key limitations include scarcity of prospective diagnostic-accuracy studies, heterogeneity in imaging indications and outcome reporting, limited pediatric-specific comparative research, and absence of robust cost-effectiveness analyses. Prospective multicenter studies are needed to refine imaging algorithms and strengthen pediatric recommendations.^59^^,^^62^

## Statement 4 ‒ Audiometric confirmation of sensorineural hearing loss

Clinicians should obtain audiometric confirmation of sensorineural hearing loss within 14-days of symptom onset in adults with suspected sudden hearing loss. Strong recommendation.

### Action statement

Quality improvement opportunity: Improve the timely and accurate diagnosis of SSNHL through objective audiometric confirmation, facilitate early treatment initiation, reduce diagnostic uncertainty, and promote adherence to evidence-based clinical pathways.

Aggregate evidence quality: Grade B, based on clinical practice guidelines, systematic reviews, cohort studies, and prognostic studies consistently demonstrating the importance of audiometric confirmation for diagnosis, treatment planning, prognostic assessment, and monitoring of hearing recovery in patients with suspected SSNHL.

Level of confidence in the evidence: High.

Benefits: Confirm the diagnosis of sensorineural hearing loss; distinguish SSNHL from conductive and mixed hearing loss; establish baseline hearing thresholds; facilitate prompt initiation of appropriate treatment; identify prognostic factors associated with hearing recovery; guide additional diagnostic investigations; monitor treatment response; improve patient counseling and expectation management; support standardized clinical decision-making.

Risks, harms, costs: Costs associated with audiologic evaluation; potential delays in diagnosis when audiometric services are not readily available; possible logistical barriers in underserved regions. No significant clinical harms are associated with audiometric testing itself.

Benefits-harm assessment: Preponderance of benefit over harm.

Value judgments: The guideline panel placed a high value on objective confirmation of hearing loss before establishing the diagnosis of SSNHL. Although clinical history may strongly suggest SSNHL, audiometric confirmation remains essential for accurate classification, exclusion of alternative diagnoses, determination of severity, assessment of prognosis, and monitoring of treatment outcomes. Early confirmation is considered critical because therapeutic efficacy may be time dependent. Treatment should not be delayed while awaiting audiometric confirmation when clinical suspicion is high.

Intentional vagueness: The statement does not mandate a specific audiometric protocol beyond obtaining formal audiometric confirmation. Alternative validated hearing assessment methods, including tele-audiology and smartphone-based audiometry, may be considered in selected circumstances when conventional audiometry is not immediately available, although pure-tone audiometry performed in a calibrated clinical environment remains the reference standard.

Role of patient preferences: Limited. Patients may participate in decisions regarding the location and timing of audiologic testing when multiple options are available; however, audiometric confirmation is considered an essential component of appropriate evaluation and management.

Exceptions: Patients who are unable to undergo conventional audiometric testing because of severe medical instability, cognitive limitations, or other exceptional circumstances may require alternative hearing assessment methods.

Policy level: Strong recommendation.

Differences of opinion: None.

**Supporting text**

Sudden Sensorineural Hearing Loss (SSNHL) is a clinical condition that requires objective audiometric confirmation to differentiate it from conductive or mixed hearing loss and to exclude alternative etiologies. Although clinical history may strongly suggest SSNHL, symptoms alone are insufficient for accurate classification, particularly in patients presenting with ear fullness or subjective hearing fluctuation.

Early audiometric assessment not only confirms the diagnosis but also establishes a baseline for prognosis and therapeutic monitoring.^63^^,^^64^^,^^66^

Current clinical practice guidelines strongly recommend obtaining audiometric confirmation within 14-days of symptom onset.^3^ Early confirmation is associated with timely treatment initiation, particularly regarding corticosteroid therapy and potential salvage interventions.^3^^,^^65^^,^^68^

Prognostic studies consistently demonstrate that the initial audiometric configuration, degree of hearing loss, and early post-treatment audiometry are significant predictors of hearing recovery.^70^^,^^71^, ^72^, ^73^ Delayed confirmation may result in missed therapeutic windows, inappropriate management, and poorer long-term audiological outcomes.^67^^,^^69^

Furthermore, audiometric evaluation plays a crucial role in identifying red flags that may suggest alternative diagnoses, such as vestibular schwannoma or other retrocochlear pathologies.^74^ In this context, audiometry also guides the need for additional diagnostic imaging.

Emerging evidence on telemedicine and validated smartphone-based audiometry suggests that remote screening may facilitate earlier confirmation in selected settings; however, standard pure-tone audiometry performed in a calibrated clinical environment remains the reference standard.^76^, ^77^, ^78^

Therefore, timely audiometric confirmation represents an essential step in the management pathway of adults with suspected sudden hearing loss and supports the strong recommendation provided in this statement.

## Methods

### Reviewed databases

A systematic search was conducted in the following electronic bibliographic databases: MEDLINE/PubMed; Embase; Web of Science; Cumulative Index to Nursing and Allied Health Literature (CINAHL); Cochrane Library; National Guideline Clearinghouse; Canadian Medical Association Infobase; and the National Institute for Health and Care Excellence (NICE).

To ensure the inclusion of contemporary evidence, only studies published from 2000 onwards and written in English were considered. Systematic reviews randomized controlled trials, prospective and retrospective cohort studies, and clinical practice guidelines were included.

The search strategy was based on the combination of the following MeSH terms: “sudden hearing loss” AND “audiometry tests” AND “diagnosis.”

### Protocol and registration

This systematic review followed the Preferred Reporting Items for Systematic Reviews and Meta-Analyses (PRISMA) guidelines.

### Eligibility criteria

This systematic review was structured according to the PICOS framework:

• Population: Adults (≥ 18-years) with a diagnostic hypothesis of sudden hearing loss.
• Intervention: Audiometry, tympanometry, and otoacoustic emissions.
• Comparison: Patients with suspected sudden hearing loss who did not undergo audiometric evaluation.
• Primary outcome: Diagnosis confirmation and treatment initiation within 14-days of symptom onset.
• Secondary outcome: Audiological follow-up 11-weeks after treatment initiation.
• Study designs: Systematic reviews, randomized controlled trials, prospective and retrospective cohort studies, and clinical guidelines.

Inclusion criteria: Adults (≥ 18-years) investigated for sudden hearing loss using audiometric testing.

Exclusion criteria: Articles without full-text access; studies classified as having a high risk of bias according to the Joanna Briggs Institute (JBI) critical appraisal tools.

### Risk of bias assessment

Studies meeting the inclusion criteria were independently evaluated by two reviewers. Risk of bias was assessed using the Joanna Briggs Institute (JBI) Critical Appraisal Checklists for Systematic Reviews, Randomized Controlled Trials, and Cohort Studies.

## Results

### Search and selection strategy

The search identified a total of 161 articles. No additional eligible studies were identified through manual reference screening. Flow diagram of the study selection process following PRISMA methodology: Identification (n = 161), Screening (n = 108), Eligibility (n = 30), Included (n = 26). A total of 26 studies were included in the qualitative synthesis.

## Statement 5 ‒ Investigation of retrocochlear pathologies

Clinicians should evaluate patients with SSNHL for retrocochlear pathology using Magnetic Resonance Imaging (MRI). Auditory Brainstem Response (ABR) may be considered only when MRI is unavailable or contraindicated. Strong recommendation.

### Action Statement

Quality improvement opportunity: Improve detection of retrocochlear pathology in patients with SSNHL through appropriate use of diagnostic imaging, facilitate early diagnosis of vestibular schwannoma and other clinically significant lesions, and reduce reliance on diagnostic strategies with insufficient sensitivity.

Aggregate evidence quality: Grade B, based on clinical practice guidelines, systematic reviews, diagnostic accuracy studies, observational cohorts, and consistent evidence demonstrating superior diagnostic performance of MRI compared with ABR for the detection of retrocochlear pathology.

Level of confidence in the evidence: High.

Benefits: Improve detection of vestibular schwannoma and other retrocochlear lesions; facilitate early diagnosis of potentially treatable pathologies; provide accurate anatomical characterization of lesions; improve clinical decision-making and patient counseling; reduce false reassurance associated with normal ABR findings; support appropriate long-term surveillance and management strategies.

Risks, harms, costs: Increased healthcare costs associated with MRI; limited MRI availability in some regions; patient discomfort related to claustrophobia or contrast administration when required; potential incidental findings leading to additional investigations. Restricting ABR use may increase dependence on advanced imaging resources.

Benefits-harm assessment: Preponderance of benefit over harm.

Value judgments: The guideline panel placed a high value on maximizing diagnostic sensitivity for retrocochlear pathology, recognizing that vestibular schwannoma and other intracranial lesions may initially present as SSNHL. Although the prevalence of these lesions is relatively low, the potential consequences of missed diagnoses were considered sufficiently important to justify routine investigation with MRI. The panel also considered the consistently superior diagnostic performance of MRI compared with ABR.

Intentional vagueness: The statement does not specify the optimal timing of MRI acquisition after diagnosis of SSNHL, nor does it mandate a particular MRI protocol beyond appropriate evaluation of the internal auditory canals and cerebellopontine angle. The role of ABR may vary according to local resource availability and patient-specific factors.

Role of patient preferences: Limited. Patients may participate in decisions regarding imaging timing and modality when clinically appropriate; however, investigation of retrocochlear pathology is considered an essential component of evidence-based evaluation of SSNHL.

Exceptions: Patients with contraindications to MRI, including certain implanted medical devices, severe claustrophobia not amenable to management, inability to tolerate MRI examination, or lack of MRI availability. In such circumstances, ABR may be considered as an alternative screening modality, recognizing its diagnostic limitations.

Policy level: Strong recommendation for MRI evaluation of retrocochlear pathology in adults with SSNHL; recommendation against using ABR as a substitute for MRI when MRI is available.

Differences of opinion: Minor disagreement existed regarding the cost-effectiveness of routine MRI in all patients with SSNHL given the relatively low prevalence of retrocochlear lesions. However, the panel reached consensus that the clinical consequences of missed diagnoses outweighed potential cost concerns.

**Supporting tex**t

## Introduction

Sudden Sensorineural Hearing Loss (SSNHL) is considered an otologic emergency, most often idiopathic, but it may represent the initial manifestation of retrocochlear pathologies, particularly vestibular schwannoma.^81^, ^82^, ^83^

Historically, the need to investigate such lesions was consolidated in clinical guidelines starting in the 2010s, notably with the American Academy of Otolaryngology-Head and Neck Surgery (AAO-HNS) guideline, updated in 2019.^3^ In this context, two main diagnostic modalities are used: Magnetic Resonance Imaging (MRI), regarded as the gold standard, and Auditory Brainstem Response (ABR), traditionally used as a screening test.^79^

Despite widespread use, the universal need for MRI and the role of ABR as an alternative or triage tool remain debated.^79^^,^^80^ This systematic review aims to synthesize evidence published between January 2015 and June 2025 addressing this issue.

## Methods

### Search strategy

A structured search was conducted using the following descriptors and keywords: sudden sensorineural hearing loss, retrocochlear pathology, vestibular schwannoma, magnetic resonance imaging, and auditory brainstem response, combined using Boolean operators.

The following databases were considered: MEDLINE (via PubMed), EMBASE, Web of Science, CINAHL, Cochrane Database of Systematic Reviews, National Guideline Clearinghouse, AMED, Canadian Medical Association Infobase, and NICE.

### Inclusion criteria

Studies published between January 2015 and June 2025. Adult population (>18-years). Randomized clinical trials, prospective or relevant retrospective observational studies, and systematic reviews. Evaluation of MRI and/or ABR in investigating retrocochlear pathology in SSNHL.

### Exclusion criteria

Pediatric studies, case reports and studies lacking imaging confirmation or adequate follow-up.

### Outcomes assessed

Detection of vestibular schwannoma or other retrocochlear lesions, sensitivity and specificity of diagnostic methods and clinical impact of diagnostic strategies.

## Results

### General characteristics of included studies

Seven high-impact publications were included, comprising clinical guidelines, systematic reviews, and large observational studies (total sample >15,000 patients). No randomized clinical trials directly comparing MRI versus ABR for this indication were identified.

### Prevalence of retrocochlear pathology

The prevalence of vestibular schwannoma among patients with SSNHL was consistently low but clinically relevant^80^^,^^82^^,^^83^:

* 1.1% to 4.5% in observational studies.
* 3.0%–3.66% in the most robust analyses.
* Lesions were typically small and early-stage.

### Diagnostic performance of magnetic resonance imaging

MRI demonstrated:

* High sensitivity for lesions of the internal auditory canal and cerebellopontine angle;
* Ability to detect multiple etiologies beyond schwannoma;
* Established role as the diagnostic gold standard.^3^^,^^80^^,^^81^

The most recent systematic review (CNS, 2025) reported a diagnostic yield of 3.66% for vestibular schwannoma in SSNHL patients.^80^

### Diagnostic performance of Auditory Brainstem Response (ABR)

Studies demonstrated^79^:

* Variable sensitivity (37%–100%)
* Specificity ranging from 57% to 96%
* Clinically significant false negatives

Key findings:

* A normal ABR does not exclude vestibular schwannoma;
* Hearing recovery does not exclude retrocochlear pathology^84^;
* Overall inconsistent diagnostic performance compared to MRI.

### Clinical and economic impact studies

Cost-effectiveness analyses showed:

* ABR → MRI strategies produce minimal cost savings;
* Approximately 2% of relevant diagnoses may be missed;
* The small economic benefit does not outweigh clinical risks.^79^

## Discussion

This review demonstrates that although the prevalence of retrocochlear lesions in SSNHL is relatively low, their detection carries substantial clinical importance. Vestibular schwannoma may initially present as sudden hearing loss, often at an early and potentially treatable stage.^82^^,^^83^

MRI remains the most reliable diagnostic method, offering superior detection and anatomical characterization.^3^^,^^80^^,^^81^ In contrast, ABR presents important limitations, including variability in diagnostic accuracy and the presence of false negatives.^79^

A critical point is that clinical improvement does not exclude structural pathology. This has direct implications for clinical practice, where imaging is sometimes deferred following hearing recovery.^84^

Although the absence of randomized trials limits the level of evidence, consistency across observational studies and guidelines strengthens the recommendations.

### Conclusion

Evidence published between 2015 and 2025 supports that magnetic resonance imaging should be the primary diagnostic modality for investigating retrocochlear pathology in adults with sudden sensorineural hearing loss. Despite the relatively low prevalence of detectable lesions, their clinical significance justifies routine MRI use.

Auditory brainstem response has limited utility and should not replace MRI when it is available. Its role should be restricted to specific clinical scenarios.

## Statement 6 ‒ Laboratory testing in sudden sensorineural hearing loss

Clinicians should not obtain routine laboratory testing in patients with SSNHL. Laboratory evaluation may be considered when guided by clinical suspicion of a specific underlying etiology. Recommendation against routine testing.

### Action Statement

Quality improvement opportunity: Reduce unnecessary laboratory investigations in patients with SSNHL, promote evidence-based diagnostic evaluation, decrease healthcare costs, and encourage targeted testing based on clinical presentation and suspected underlying etiologies.

Aggregate evidence quality: Grade B, based on systematic reviews, observational studies, and meta-analyses demonstrating inconsistent associations between laboratory abnormalities and clinical outcomes, with insufficient evidence that routine laboratory testing improves diagnosis, treatment, prognosis, or patient-centered outcomes.

Level of confidence in the evidence: High.

Benefits: Reduce unnecessary testing; decrease healthcare costs; minimize false-positive results and incidental findings; avoid additional investigations of uncertain clinical value; encourage focused etiologic evaluation when clinically indicated; improve resource utilization; simplify diagnostic pathways.

Risks, harms, costs: Potential failure to identify uncommon systemic disorders in patients undergoing limited laboratory evaluation; delayed diagnosis of selected infectious, autoimmune, hematologic, or metabolic conditions if clinical suspicion is not recognized. These risks can be minimized through careful history-taking and targeted laboratory investigation when indicated.

Benefits-harm assessment: Preponderance of benefit over harm.

Value judgments: The guideline panel recognized that several laboratory biomarkers, particularly inflammatory markers, coagulation parameters, and hematologic indices, have demonstrated potential prognostic associations with SSNHL outcomes. However, the available evidence does not demonstrate that routine laboratory screening changes clinical management, improves hearing recovery, or increases diagnostic yield sufficiently to justify universal testing. The panel placed greater value on targeted testing directed by clinical findings and suspected etiologies.

Intentional vagueness: The statement does not specify which laboratory tests should be obtained when clinically indicated. Selection of laboratory investigations should be individualized according to patient history, physical examination findings, comorbidities, risk factors, and suspected infectious, autoimmune, vascular, metabolic, or hematologic disorders.

Role of patient preferences: Moderate. Patients may wish to pursue broader diagnostic investigations despite limited evidence supporting routine laboratory testing. Shared decision-making may be appropriate when considering targeted laboratory evaluation in the absence of clear clinical indications.

Exceptions: Patients with clinical findings suggestive of autoimmune disease, systemic inflammatory disorders, infectious diseases, hypercoagulable states, hematologic disorders, metabolic abnormalities, vasculitis, bilateral SSNHL, recurrent SSNHL, fluctuating hearing loss, or other atypical presentations.

Policy level: Recommendation against routine laboratory testing.

Differences of opinion: Minor disagreement existed regarding the potential prognostic value of inflammatory, coagulation, and hematologic biomarkers. Some panel members favored broader laboratory screening because of emerging evidence suggesting prognostic associations; however, consensus was reached that current evidence does not support routine testing in all patients with SSNHL.

**Supporting Tex**t

## Evidence synthesis

Overall, the studies were heterogeneous with respect to laboratory tests evaluated, methodological design, and outcomes measured. Laboratory investigations most frequently assessed included complete blood count, inflammatory markers, autoimmune and infectious serologies, lipid profile, coagulation parameters, and vitamins.

When considering the prognostic role of laboratory testing in SSNHL, the evidence was inconsistent. Several observational studies reported associations between elevated inflammatory markers, abnormal coagulation profiles, or metabolic alterations and poor hearing recovery.

Twenty-two^85^, ^86^, ^87^, ^88^, ^89^, ^90^, ^91^, ^92^, ^93^, ^94^, ^95^, ^96^, ^97^, ^98^, ^99^, ^100^, ^101^, ^102^, ^103^, ^104^, ^105^ of the 33 studies (66.67%) tend to support performing some kind of laboratory testing as routine, arguing its prognostic value.

However, other 11 studies^3^^,^^106^, ^107^, ^108^, ^109^, ^110^, ^111^, ^112^, ^113^, ^114^ (33.33%) did not replicate these findings, and most were limited by small sample size, retrospective design, or lack of standardized testing protocols.

Randomized or quasi-experimental studies directly comparing patients who underwent laboratory testing versus those who did not were scarce. Instead, most publications investigated correlations between individual biomarkers and hearing outcomes, without clear demonstration that routine testing improved patient management or prognosis.

Furthermore, systematic reviews and meta-analyses addressing the topic highlighted the prognostic value, especially of inflammatory markers and fibrinogen.^97^^,^^98^^,^^100^^,^^102^^,^^103^

However, they pointed out the same limitations, underscoring the absence of robust, high-quality evidence to support routine broad laboratory screening in all SSNHL patients.

From a pathophysiological standpoint, cochlear vascular microthrombosis has been hypothesized as a pathogenic mechanism, involving thrombophilia or endothelial dysfunction.^95^ However, usual thrombophilia screening has previously led to conflicting results.

A concurrent hypothesis proposed that SSNHL does not result from thrombophilic abnormalities but rather a vasospasm.^95^ Serotonin is a well-known neurotransmitter that is also produced peripherally by non-neuronal cells in the gut called the enterochromaffin cells. The majority of peripheral serotonin is stored in platelet dense granules and is released from the platelets in response to various signals, including damaged endothelium or ischemia.

Once released, serotonin exhibits a strong vasoconstrictive action that can lead to vasospasm.^95^ Study conducted with 133 subjects found that increased plasma serotonin appears as a biomarker of SSNHL (specificity: 96%, sensitivity: 90%) and could participate in the pathophysiology of SSNHL.^95^

Immunomodulated Inner Ear Disease (IMIED) is another entity whose clinical manifestations (hearing loss, tinnitus and vertigo) are shared with different etiologies (i.e., vascular, viral, toxic, metabolic, genetic or idiopathic).^90^

The diagnosis of IMIED represents a challenge, particularly in isolated forms in which there are no systemic manifestations to support the diagnosis. However, there is no specific marker to this point that can identify IMIED.

Some antibodies such as anti-heat shock protein (anti-HSP-70; 68-kDa protein) have been described, but they have low sensitivity and specificity levels.^115^ Targeted testing (ANA and immunophenotype of PBL) can be more helpful.^90^

Another topic worth mentioning is that although the diagnosis of SSNHL is based upon clinical and audiometric criteria, sometimes previous audiometry is unavailable, and the clinical history may be unclear.

The results of a recent meta-analysis showed that high NLR, PLR, and CRP/Alb indicated the occurrence of SSNHL, and that NLR and PLR could predict prognosis of SSNHL.^98^ Despite these findings, no biomarker has yet been validated for routine clinical use or shown to meaningfully influence clinical decision-making or therapeutic management.

In terms of treatment, laboratory tests did not change clinical management. However, understanding the underlying metabolic disorders can take us one step closer to discovering the real etiology of idiopathic SSNHL, and this can have a real impact on the management and prognosis of these patients for the future.

### Conclusion

Based on the synthesis of 30 studies, there is no strong evidence that routine laboratory testing in SSNHL consistently improves diagnostic yield, prognostic accuracy, or patient care.

Some laboratory parameters, particularly inflammatory and coagulation markers, may have prognostic value in selected patients, but findings remain inconclusive.

Although several biomarkers have demonstrated prognostic associations, there is no evidence that routine laboratory testing improves treatment decisions or hearing outcomes. Laboratory evaluation should therefore be guided by the individual clinical context and suspected etiologies.

Further large-scale, prospective, and standardized studies are warranted to clarify the role of laboratory investigations in the etiological work-up and prognosis of SSNHL.

## Statement 7 ‒ The role of genetic evaluation in sudden sensorineural hearing loss

Clinicians should not routinely obtain genetic testing in patients with SSNHL. Genetic evaluation may be considered in selected patients with bilateral SSNHL, recurrent episodes, family history of hearing loss, pediatric presentation, or other clinical features suggestive of a hereditary predisposition. Recommendation against routine testing.

### Action statement

Quality improvement opportunity: Promote appropriate utilization of genetic testing in SSNHL, reduce unnecessary investigations, improve identification of patients with potential hereditary susceptibility, and facilitate personalized diagnostic evaluation in selected clinical scenarios.

Aggregate evidence quality: Grade C, based on observational studies, systematic reviews, genetic association studies, and limited sequencing studies demonstrating potential associations between genetic variants and SSNHL susceptibility or prognosis, but lacking validation across populations and evidence of clinical utility in routine practice.

Level of confidence in the evidence: Moderate.

Benefits: Identify patients with possible genetic susceptibility to SSNHL; facilitate genetic counseling; improve understanding of disease pathophysiology; support prognostic assessment in selected patients; identify individuals who may benefit from future precision medicine approaches; guide family counseling regarding inheritance patterns; reduce diagnostic uncertainty in atypical or recurrent cases.

Risks, harms, costs: High costs of genetic testing; limited availability in many healthcare systems; identification of Variants of Uncertain Significance (VUS); risk of overinterpretation of findings with uncertain clinical relevance; potential patient anxiety; limited impact on current therapeutic decision-making.

Benefits-harm assessment: Balance of benefits and harms for routine testing; preponderance of benefit over harm in selected high-risk patients.

Value judgments: The guideline panel recognized the growing evidence supporting a genetic contribution to SSNHL susceptibility and prognosis. However, the current literature is characterized by methodological heterogeneity, lack of replication of most identified variants, population-specific findings, and limited demonstration of direct clinical utility. The panel placed greater value on targeted genetic evaluation in selected patients than on routine testing in all individuals with SSNHL.

Intentional vagueness: The statement does not specify a mandatory genetic testing strategy or panel composition. The choice of genetic investigation should be individualized according to clinical presentation, family history, age at presentation, recurrence pattern, and local availability of genetic services.

Role of patient preferences: Moderate. Patients may place substantial value on obtaining a potential etiologic diagnosis, understanding hereditary risk, and receiving genetic counseling. Shared decision-making is appropriate when considering genetic testing in selected cases.

Exceptions: Patients with bilateral SSNHL, recurrent SSNHL, positive family history of hearing loss, pediatric onset, young patients without traditional vascular risk factors, incomplete recovery, steroid-resistant disease, or clinical features suggestive of hereditary hearing disorders.

Policy level: Recommendation against routine genetic testing; option for selective genetic evaluation in appropriately selected patients.

Differences of opinion: Minor disagreement existed regarding the threshold for recommending genetic testing in adults with apparently idiopathic SSNHL. Some panel members favored broader use of genetic panels because of emerging evidence linking specific variants to disease susceptibility and prognosis. However, consensus was reached that current evidence remains insufficient to support routine testing in all patients.

**Supporting text**

## Discussion

Advances in genetics in hearing loss investigation in recent years have enabled genetic panels to play a prominent role in the evaluation of cases of hearing loss that were previously considered idiopathic. In the case of sudden hearing loss, the risk of recurrence among siblings supports a significant genetic contribution. However, although several genetic variants have been reported as associated with sudden hearing loss in controlled studies, none have been replicated in an independent cohort.^116^, ^117^, ^118^, ^119^

Considering that its pathogenesis appears to be multifactorial ‒ i.e., associated with viral infections, immune-mediated diseases, and microcirculatory impairment together with the heterogeneity of genetic studies reported in the literature, there is no evidence that standardizing genetic investigation for all cases of sudden hearing loss provides benefits. The costs of genetic panels, combined with limited availability in underdeveloped and developing countries, makes this scenario even more remote as a routine approach for all cases.^120^, ^121^, ^122^, ^123^

Nevertheless, it is evident that genetic susceptibility in sudden hearing loss plays a key role in its pathogenesis.^124^^,^^125^ In cases with a family history of sudden hearing loss, bilateral involvement, sudden hearing loss in children, lack of improvement or response to corticosteroid treatment, recurrent sudden hearing loss, and idiopathic cases especially in young individuals without vascular risk factors genetic evaluation adds diagnostic and prognostic value.^116^^,^^117^^,^^126^^,^^127^

Identifying a potential genetic profile related to sudden hearing loss allows genetic counseling to provide information regarding hearing-loss prognosis and future gene therapy or alternative treatment modalities. In addition, early prevention strategies can be outlined, together with counseling about inheritance patterns of hearing loss. Reducing patient frustration and anxiety by establishing a diagnosis is also a benefit of genetic investigation.^117^

Several genetic polymorphisms related to oxidative stress, inflammation, thrombosis, and vascular permeability are associated with predisposition to and development of sudden hearing loss. Therefore, genetic factors play a role in maintaining the delicate balance between free radicals and antioxidants; pro-inflammatory and anti-inflammatory factors; and pro-aggregatory and anti-aggregatory factors. Inner ear homeostasis depends on a delicate balance, disruption of which contributes to the development of sudden hearing loss.^128^, ^129^, ^130^, ^131^

Genes such as Superoxide Dismutase 1 (SOD1) and factor V Leiden have been associated as possible factors involved in the pathogenesis of sudden hearing loss. Superoxide dismutase is an important antioxidant system that protects inner ear cells by providing protection against free radicals and ototoxic substances. Specific SOD1 polymorphisms, such as SOD1 rs4998557, appear to be associated with sudden hearing loss due to loss of an efficient antioxidant mechanism.^131^^,^^132^

In the case of factor V Leiden, the mutation results in a hypercoagulable state and thromboembolic disorders.^132^, ^133^, ^134^

Polymorphisms in inflammation-related genes have been widely investigated, as activation of intracellular oxidative pathways in the inner ear neuroepithelium by inflammatory cytokines disrupts intracochlear homeostasis, causing sudden hearing loss. While interleukins are cytokines involved in activation and regulation of the immune system, Intercellular Adhesion Molecules (ICAM-1) are associated with the magnitude of the inflammatory response.

The combined presence of the IL-6 c.572C > G and ICAM-1 p.Lys469Glu polymorphisms is significantly related to an increased risk of sudden sensorineural hearing loss.^135^, ^136^, ^137^, ^138^, ^139^, ^140^

The systemic pathogenesis of sudden hearing loss allows a connection with cardiovascular disease, especially in cases involving polymorphisms that affect pro-aggregatory and anti-aggregatory homeostasis. Investigation of personal history of stroke and ischemic cardiovascular disease becomes indispensable in this context.

Assessment of family medical history for ischemic disease, degree of kinship, and phenotype should always be sought as a possible diagnostic tool, as well as other cardiovascular risk factors in patients affected by sudden hearing loss.^135^, ^136^, ^137^, ^138^, ^139^, ^140^, ^141^, ^142^

Genetic polymorphisms have also been shown to be related to prognosis of hearing recovery, especially after corticosteroid therapy. Some genetic factors may confer resistance to corticosteroid treatment, and identifying these genes and assessing prognosis may allow selection of personalized therapy, i.e., a therapeutic strategy fully tailored after establishing a genetic diagnosis.^143^^,^^144^

Some oxidative stress-related genes, such as Glutathione Reductase (GSR) rs2251780 and rs3779647, and Nitric Oxide Synthase 3 (NOS3) rs1799983, are associated with an unfavorable outcome with corticosteroids. In this context, other therapies, such as hyperbaric oxygen therapy or intratympanic corticosteroid administration, may be modalities to be considered earlier as initial or salvage therapy.^116^^,^^117^^,^^143^^,^^144^

Despite advances in genetics in the field of hearing loss, most studies available in the literature involve small samples, evaluate specific ethnic groups, and additional studies are needed to better understand the correlation between sudden hearing loss and genetic polymorphisms.

At the same time, there are only small studies or case reports correlating phenotypes compatible with sudden hearing loss and genetic mutations associated with syndromic and non-syndromic hereditary hearing loss.^124^, ^125^, ^126^, ^127^

Additional studies will allow more precise identification of reliable genetic risk factors associated with sudden hearing loss. Recognizing patients with genetic risk factors for sudden hearing loss may be advantageous in planning preventive measures and therapeutic intervention strategies.^145^^,^^146^

### Clinical implications and recommendation

Genetic evaluation is not routinely recommended in all cases of SSNHL. However, there are specific scenarios in which ordering it is justified:

• Bilateral sudden hearing loss;
• Sudden hearing loss in children
• Presence of a family history of hearing loss;
• Recurrent SSNHL cases;
• Young individuals without vascular risk factors;
• Lack of therapeutic response or incomplete recovery.

In these cases, it is recommended to start with multigene panels targeted to genes related to acquired hearing loss (e.g., SOD2, GSR, NOS3, IL6, FCRL3, STRC, GJB2). When results are negative and there is strong clinical suspicion, WES (exome) or WGS (genome) may be considered, preferably in a research setting.^116^^,^^117^^,^^126^^,^^127^

### Conclusion

Genetic evaluation in patients with SSNHL shows potential to identify variants associated with susceptibility and hearing prognosis. Although it has not yet been broadly integrated into clinical practice, genetic screening should be considered in patients with a family history, bilateral sudden hearing loss, or recurrent episodes.

Future research should employ standardized methodologies, include control groups, and consider validated genetic panels to increase the clinical applicability of these findings.

## Statement 8 ‒ CORTICOSTEROID THERAPY

Clinicians should offer corticosteroid therapy as initial treatment to patients with SSNHL within two weeks of symptom onset. Systemic corticosteroids, intratympanic corticosteroids, or both may be considered according to clinical circumstances and patient-specific factors. Strong recommendation.

### Action statement

Quality improvement opportunity: Promote timely initiation of evidence-based treatment for SSNHL, maximize the likelihood of hearing recovery, reduce variation in clinical practice, and improve patient counseling regarding therapeutic options and expected outcomes.

Aggregate evidence quality: Grade B, based on clinical practice guidelines, randomized controlled trials with methodological limitations, systematic reviews, and meta-analyses demonstrating potential benefit of corticosteroid therapy in improving hearing outcomes when administered during the acute phase of SSNHL.

Level of confidence in the evidence: High.

Benefits: Potential improvement in hearing recovery; preservation of auditory function; reduction of long-term hearing disability; improvement in quality of life; availability of multiple therapeutic routes; possibility of treatment in patients with contraindications to systemic therapy through intratympanic administration; potential additional benefit of combined systemic and intratympanic therapy in selected patients with severe hearing loss.

Risks, harms, costs: Systemic corticosteroid adverse effects including hyperglycemia, hypertension, gastrointestinal complications, mood changes, immunosuppression, weight gain, glaucoma, and osteoporosis; discomfort associated with intratympanic injections; risk of transient dizziness, pain, tympanic membrane perforation, or otitis media after intratympanic treatment; treatment-related costs and need for follow-up.

Benefits-harm assessment: Preponderance of benefit over harm.

Value judgments: The guideline panel recognized that the available evidence is limited by heterogeneity in diagnostic criteria, treatment protocols, outcome definitions, and study quality. Nevertheless, corticosteroids remain the most widely accepted and extensively studied treatment modality for SSNHL. Given the potential for permanent hearing impairment and the relative safety of treatment when appropriately prescribed, the panel placed substantial value on offering corticosteroid therapy during the acute phase of the disease.

Intentional vagueness: The statement does not mandate a specific corticosteroid agent, route of administration, concentration, treatment duration, or dosing regimen. Selection of systemic therapy, intratympanic therapy, or combined treatment should be individualized according to hearing loss severity, patient comorbidities, contraindications, treatment availability, and clinician judgment.

Role of patient preferences: Moderate. Patients may reasonably choose among systemic, intratympanic, or combined treatment approaches after discussion of the potential benefits, risks, adverse effects, convenience, and costs associated with each modality.

Exceptions: Patients presenting beyond the therapeutic window, patients with absolute contraindications to corticosteroid therapy, patients who decline treatment after informed discussion, or patients with alternative diagnoses requiring different management.

Policy level: Strong recommendation.

Differences of opinion: Minor disagreement existed regarding the magnitude of benefit attributable to corticosteroid therapy and the superiority of specific treatment routes. Some panel members emphasized the limitations of the available evidence, whereas others favored treatment based on clinical experience and the absence of more effective alternatives. Consensus was reached that corticosteroid therapy should remain the cornerstone of initial management for SSNHL.

**Supporting text**

Regarding Sudden Sensorineural Hearing Loss (SSNHL), issues such as the most effective initial treatment, therapeutic dose, and treatment duration are still conflicting in the literature, or even questioned regarding their necessity, and lack robust scientific evidence.^3^^,^^147^ Furthermore, many studies do not standardize the criteria used to define the diagnosis of Sudden Sensorineural Hearing Loss (SSNHL), as well as present criteria for post-treatment improvement also without a standardized definition, demonstrating the heterogeneity of the studies.^147^

Currently, several guidelines propose that acute phase treatment (with a period of symptom onset within 2-weeks) may consist primarily of corticosteroid therapy, whether performed with oral corticosteroids, Intravenous (IV) corticosteroids, Intratympanic (IT) corticosteroids, or even combined therapy with simultaneous oral and intratympanic corticosteroids.^3^^,^^147^, ^148^, ^149^ Some studies also evaluate and propose initial corticosteroid therapy with the patient inpatient or outpatient, depending on the severity of SSNHL.

Oral corticosteroid therapy is generally proposed as first-line treatment based on an assessment of the risk-benefit ratio.^3^^,^^147^ Potential risks of using oral systemic corticosteroid therapy include: acne, worsening of diabetes, hypertension, decreased resistance to infections, muscle weakness, osteoporosis, cataracts or glaucoma, weight gain, among others.^3^^,^^149^

Chandrasekhar et al., in their latest update of the American Academy of Otolaryngology-Head & Neck Surgery (AAO-HNS) guideline, and the latest Cochrane review, analyzed three eligible Randomized Controlled Trials (RCTs) evaluating the use of oral corticosteroids versus placebo in the initial treatment of SSNHL. They concluded that oral corticosteroids were either not proven effective or ineffective due to the high risk of bias. Only one of these RCTs showed hearing improvement in 61% of patients in the oral corticosteroid group compared with 32% in the control group.^3^^,^^147^^,^^148^

Based on the studies presented, the otolaryngologist may choose not to prescribe oral corticosteroids to the patient. However, due to the potential risk of permanent hearing impairment, with potential worsening of quality of life, treatment with oral corticosteroids is one of the few treatment options with data demonstrating efficacy, although these data are biased.^3^^,^^147^^,^^148^

When oral corticosteroid therapy was used, it offered greater recovery when initiated within the first two weeks of symptom onset.^3^ The corticosteroids used are prednisolone, methylprednisolone, and dexamethasone.

The recommended dose of prednisolone is 1 mg/kg/day in a single dose, with a maximum dose of 60 mg/day, with treatment duration ranging from 10 to 14 days without the need for de-escalation.^3^^,^^147^ If methylprednisolone or dexamethasone is used, their respective doses equivalent to 60 mg of prednisolone are: 48 mg and 10 mg.^3^

Eftekharian et al., based on a randomized clinical trial comparing high doses of corticosteroids versus the usual dose, studied 67 patients with SSNHL, who were divided into two groups.^150^

The study group received 500 mg of intravenous methylprednisolone daily for 3 consecutive days, followed by 1 mg/kg/day (maximum of 60 mg) of oral prednisolone for 11-days (total treatment: 14-days); the control group received 1 mg/kg/day (maximum of 60 mg) of oral prednisolone for 14-days.^150^

Sixty of the 67 patients (29 of the 34 patients in the study group and 31 of the 33 patients in the control group) completed the study. The frequency-specific hearing improvement did not differ significantly between the groups. The improvement in speech recognition score was 20.34%±27.35% for the study group and 13.41%±23.48% for the control group, with no statistically significant difference. There was also no significant difference in the hearing recovery rates between the two groups.^150^

However, if the patient with SSNHL has a comorbidity that contraindicates the use of oral corticosteroids, such as insulin-dependent or poorly controlled diabetes, labile hypertension, glaucoma, tuberculosis, or peptic ulcer, the use of intratympanic corticosteroid injections can be considered without inferior efficacy compared with systemic treatment.^3^^,^^147^, ^148^, ^149^

Treatment with intratympanic corticosteroid injections has gained popularity since its recommendation in 2012, especially in patients with contraindications to oral corticosteroids.^3^

The main theoretical advantage of intratympanic corticosteroids is the bypass of the blood-labyrinthine barrier to achieve higher concentrations in the inner ear.^3^^,^^147^^,^^149^ However, similar to studies using oral corticosteroid therapy, the data supporting the use of intratympanic corticosteroids as initial therapy for SSNHL are ambiguous.^3^^,^^148^

Several recent meta-analyses have shown no significant difference in improved recovery rate of mean audiometric thresholds between oral and intratympanic corticosteroids when used as primary therapy.^147^

The most commonly administered intratympanic corticosteroids are dexamethasone and methylprednisolone. The concentrations used vary widely between studies; most articles report doses of 4–24 mg/mL of dexamethasone and ≥30 mg/mL of methylprednisolone.^3^

Higher concentrations appear to produce better results; however, evidence comparing different concentrations remains limited.^3^ Furthermore, the frequency of intratympanic corticosteroid administration also varies greatly between studies, with applications ranging from once daily to once weekly or less.^3^

After intratympanic corticosteroid application in the affected ear, the patient should remain at rest for 15−30 min after injection.^3^

Some studies have evaluated intratympanic administration of corticosteroids concomitantly with systemic corticosteroids as initial therapy for SSNHL.^3^^,^^147^, ^148^, ^149^ However, the efficacy of combination therapy remains controversial.^149^

Gao et al. conducted a meta-analysis in 2016 to investigate whether concomitant administration of intratympanic and systemic corticosteroids would provide an additional advantage over systemic corticosteroids alone in patients with SSNHL.^149^

Eight eligible studies were identified (seven prospective studies and one retrospective) comparing combination therapy with systemic therapy alone as initial treatment.^149^

The outcomes evaluated were divided into Complete Recovery (CR), Partial Recovery (PR), Mild Improvement (MI), and No Recovery (NR). Due to heterogeneity among studies, recovery analysis focused mainly on CR and NR.^149^

Gao et al. concluded that combined intratympanic and systemic corticosteroid therapy showed better results in improving mean audiometric thresholds compared with systemic therapy alone, especially in patients with severe-to-profound initial hearing loss (RR = 3.47; 95% CI 1.30–9.28; p = 0.01).^149^

Finally, in light of all the studies presented, initial therapy with corticosteroids, even with a small possibility of hearing improvement, appears to be a reasonable treatment option to be offered to patients with SSNHL, taking into account the damage that sensorineural hearing loss can cause and the profound impact on patients' quality of life.^3^^,^^147^

## Statement 9 ‒ Hyperbaric oxygen therapy associated with corticosteroids in sudden hearing loss as initial therapy

Clinicians may consider Hyperbaric Oxygen Therapy (HBOT) combined with corticosteroids as initial treatment in selected patients with profound SSNHL. Option.

### Action statement

Quality improvement opportunity: Improve hearing recovery in selected patients with severe or profound SSNHL through the appropriate use of adjunctive hyperbaric oxygen therapy while avoiding unnecessary treatment in patients unlikely to benefit.

Aggregate evidence quality: Grade C, based primarily on observational studies with critical risk of bias, heterogeneous treatment protocols, and limited evidence suggesting benefit in selected subgroups of patients with profound hearing loss.

Level of confidence in the evidence: Low.

Benefits: Potential improvement in hearing recovery; increased oxygen delivery to the cochlea; possible reversal of hypoxia-related cochlear injury; potential enhancement of corticosteroid treatment effects; possible improvement in functional outcomes among patients with profound hearing loss.

Risks, harms, costs: High treatment costs; limited availability of hyperbaric facilities; need for multiple treatment sessions; treatment burden for patients; risk of middle ear barotrauma, sinus barotrauma, transient visual changes, claustrophobia, and discomfort associated with hyperbaric chamber exposure.

Benefits-harm assessment: Balance of benefits and harms. Potential benefit may outweigh harms in selected patients with profound hearing loss when treatment is initiated early.

Value judgments: The guideline panel recognized the biological plausibility of improving cochlear oxygenation and the potential benefit demonstrated in some studies involving patients with profound hearing loss. However, the panel also recognized the low certainty of the available evidence, substantial methodological limitations, high treatment costs, and restricted accessibility of hyperbaric oxygen therapy.

Intentional vagueness: The statement does not define the optimal number of sessions, treatment pressure, session duration, or exact treatment initiation window beyond recommending early therapy. Clinical protocols may vary according to institutional expertise and local availability.

Role of patient preferences: High. Decisions regarding HBOT should incorporate patient preferences, considering treatment burden, availability, travel requirements, cost, potential benefits, and tolerance of hyperbaric chamber therapy.

Exceptions: Patients with untreated pneumothorax, certain pulmonary disorders, inability to tolerate hyperbaric chamber treatment, severe claustrophobia, or other contraindications to hyperbaric oxygen therapy.

Policy level: Option.

Differences of opinion: Significant disagreement existed regarding the magnitude of benefit, cost-effectiveness, and appropriate patient selection for HBOT. While some panel members favored broader use in severe hearing loss based on available observational data, others emphasized the low certainty of evidence and substantial resource requirements. Consensus was reached that HBOT may be considered in selected patients, particularly those with profound hearing loss.

**Supporting Text**

Hyperbaric Oxygen Therapy (HBOT) has been used to treat inner ear disorders since the early 1970s. Several researchers suggested that circulatory disorders were the main cause of sudden hearing loss.^151^^,^^152^ The purpose of HBOT in treating Sudden Hearing Loss (SHL) is to increase the partial pressure of oxygen in the blood and, through diffusion, increase oxygen levels in the inner ear fluids that supply the sensory and neural elements of the cochlea.

## Methodology

We included only studies that analyzed participant groups comparing initial treatment with systemic corticosteroids versus HBOT combined with systemic corticosteroids. Studies that included additional therapies in the treatment group were excluded to avoid confounding effects. Studies evaluating outcomes with less than 15-days of follow-up were also excluded.

The PICO strategy was used:

• P (Population): Adult patients (> 18-years) with sudden hearing loss (American Academy criteria: 30 dB, 3 frequencies, 3 days).
• I (Intervention): HBOT administered within 30-days of onset. Protocol: > 2.0 ATA, > 90-minutes, minimum 10 sessions.
• C (Comparison): Same treatment without HBOT (systemic/oral corticosteroids).
• O (Outcome): Improvement in hearing thresholds.

Databases searched included Medline (PubMed), LILACS (VHL), Scopus, Embase, Web of Science, and grey literature (Google Scholar, ProQuest). Search terms included MeSH, DeCS, and free terms such as “sudden deafness” AND “hyperbaric oxygenation”, with no restrictions on year or language. Study selection and evaluation were conducted independently by two reviewers, with a third reviewer resolving disagreements.

## Results

Two studies were included.^153^^,^^154^ Meta-analysis was not feasible due to heterogeneity in reported outcomes. Both were non-randomized observational studies. Risk of bias was assessed using ROBINS-I V2^156^ and classified as critical in both studies due to confounding factors:

In Tasdöven et al.,^153^ HBOT was only offered to patients who could afford it.

In Fujimura et al.,^154^ groups differed in baseline hearing loss severity.

Although both studies evaluated HBOT as initial therapy, inclusion criteria allowed treatment initiation up to 30-days^154^ and 15-days^153^ after onset.

Despite limitations, both studies showed benefit of HBOT in patients with profound hearing loss (≥ 80 dB). It could be considered an alternative for patients with more severe hearing loss, especially if they have other limitations that make sudden hearing loss an even more limiting factor, such as patients with other sensory impairments, taking into account the risks and especially the cost of treatment.

Performing an analysis exclusively for the subgroup of patients with profound sudden sensorineural hearing loss treated with hyperbaric oxygen therapy combined with corticosteroids versus corticosteroids alone reveals relevant findings, although with important limitations.

Regarding the outcome hearing response to treatment (gain >15 dB in pure tone average after approximately 15-days), the group receiving combined therapy showed a response rate of 60% (9/15), compared to 19% (4/21) in the corticosteroid-only group.

The relative risk of 3.15 (95% CI: 1.18–8.34) indicates a significant increase in the likelihood of hearing improvement with the addition of HBOT.^153^^,^^157^

However, the certainty of evidence for this outcome was classified as low, mainly due to extremely serious risk of bias and imprecision, despite the absence of inconsistency and indirectness.^155^

The presence of a strong association and the consideration that potential confounding factors would likely reduce the observed effect strengthen confidence in the direction of effect, though not its exact magnitude.

On the other hand, in the analysis of Fujimura et al.,^154^ when evaluating hearing improvement rate (continuous scale after approximately 30-days), the combined therapy group had an average improvement of 51.1 dB compared to 27.1 dB in the control group, with a mean difference of 24-points (95% CI: 18.78–29.22).

This demonstrates a clinically meaningful effect favoring HBOT combined with corticosteroids.^154^

The certainty of evidence was classified as high, despite being derived from an observational study, due to the magnitude of effect, consistency of findings, and the evaluation that potential biases would likely decrease rather than increase the observed effect.^155^

Overall, the analysis suggests that, although there are methodological limitations, the data point to a potential benefit of combined therapy in patients with profound sudden hearing loss, with a moderate recommendation strength.

The discrepancy between certainty levels highlights the need for caution in interpretation and underscores the importance of additional studies, preferably randomized controlled trials, to better define effect and standardize care.

### Recommendation

There is insufficient evidence to recommend HBOT as an initial treatment for sudden hearing loss in general. Some benefit appears to exist for cases of profound hearing loss (> 80 dB).^153^^,^^154^

### Overall justification

Two observational intervention studies with non-randomized comparison groups were selected. Both studies had critical risk of bias due to group selection issues, which may have influenced the results, leading to low reliability.

Additionally, no benefit from HBOT combined with corticosteroids was observed except in the subgroup of patients with profound hearing loss.^153^^,^^154^

Another factor to consider is the high cost of HBOT treatment.

### Detailed justification

Certainty of Evidence: Low certainty, critical risk of bias.^155^^,^^156^

Cost-effectiveness: High-cost Treatment (HBOT).

Feasibility: Difficult access, high cost, time-consuming, and limited availability.

Subgroup Considerations: Analysis restricted to patients with profound hearing loss (> 80 dB) showed benefit from HBOT.^153^^,^^154^

### Implementation considerations

Based on the findings, HBOT should not be considered first-line treatment for sudden hearing loss. Individualized cases may be considered in profound hearing loss, especially when associated with other deficits (e.g., visual impairment) or single-sided hearing loss. Cost and availability should also be considered.

## Statement 10 ‒ Intratympanic corticosteroid (salvage therapy?)

Clinicians should offer, or refer to a clinician who can offer, intratympanic corticosteroid therapy to patients with incomplete recovery from SSNHL within 2 to 6-weeks after symptom onset. Recommendation.

### Action statement

Quality improvement opportunity: Improve hearing recovery in patients with incomplete recovery after initial treatment for SSNHL by providing access to an effective salvage therapy with a favorable safety profile and limited systemic adverse effects.

Aggregate evidence quality: Grade B, based on randomized controlled trials, systematic reviews, meta-analyses, and observational studies demonstrating that intratympanic corticosteroid therapy may improve hearing outcomes compared with placebo, no treatment, or observation in patients with persistent hearing loss after initial therapy.

Level of confidence in the evidence: Moderate.

Benefits: Potential improvement in hearing recovery; direct drug delivery to the inner ear; minimal systemic corticosteroid exposure; applicability in patients with contraindications to systemic corticosteroids; favorable safety profile; potential improvement in speech discrimination and quality of life; availability as an outpatient procedure.

Risks, harms, costs: Procedure-related discomfort; transient pain; dizziness or vertigo; tympanic membrane perforation; otitis media; need for multiple office visits; procedural costs; uncertainty regarding optimal treatment protocol.

Benefits-harm assessment: Preponderance of benefit over harm.

Value judgments: The guideline panel placed substantial value on providing patients with an additional therapeutic option after incomplete recovery following initial treatment. Although the magnitude of hearing improvement is generally modest and study protocols are heterogeneous, intratympanic corticosteroid therapy offers meaningful potential benefit with relatively low risk and minimal systemic adverse effects.

Intentional vagueness: The statement does not specify the preferred corticosteroid agent, concentration, injection volume, number of injections, or administration schedule. Treatment protocols may vary according to clinician experience, patient characteristics, and institutional practice.

Role of patient preferences: Moderate. Patients may reasonably differ in their willingness to undergo office-based intratympanic injections, accept procedural discomfort, and pursue additional treatment despite the possibility of modest hearing improvement. Shared decision-making is encouraged.

Exceptions: Patients who achieve complete recovery after initial treatment, patients unable to tolerate intratympanic procedures, patients with active middle ear infection, or patients who decline additional intervention.

Policy level: Recommendation.

Differences of opinion: Minor disagreement existed regarding the magnitude of hearing improvement attributable to salvage intratympanic corticosteroid therapy and the optimal treatment protocol. However, there was broad agreement that the potential benefits outweigh the relatively low risks in appropriately selected patients.

**Supporting Text**

Therapeutic approaches for the treatment of SSNHL remains challenging, and corticosteroids continue to represent the main pharmacologic treatment option.^3^

Although systemic corticosteroids are widely used as first-line therapy, a considerable proportion of patients experience incomplete recovery or fail to respond adequately. In this context, intratympanic corticosteroid therapy has emerged as an important salvage treatment option for patients with persistent hearing loss after initial therapy.^3^^,^^158^^,^^159^

The rationale for intratympanic corticosteroid administration is based on the possibility of achieving higher inner ear drug concentrations while minimizing systemic adverse effects.^160^, ^161^, ^162^, ^163^, ^164^

Drug diffusion occurs primarily through the round window membrane, allowing corticosteroids to reach the perilymph and cochlear structures.^76^^,^^164^

Experimental pharmacokinetic studies demonstrated that local application can result in higher intracochlear concentrations compared with systemic administration, supporting its use in SSNHL.^160^

## Methods

A systematic evaluation of the literature was performed to assess the role of intratympanic corticosteroid therapy as salvage treatment for patients with SSNHL who failed to recover after initial therapy.

The review focused on studies evaluating:

• Intratympanic corticosteroid administration after incomplete recovery with systemic therapy;
• Hearing outcomes after salvage therapy;
• Timing of intervention;
• Different corticosteroid agents, concentrations, and administration protocols.

Available evidence included randomized controlled trials, prospective studies, retrospective cohorts, and systematic reviews.^158^^,^^159^

## Results

Several studies have evaluated the efficacy of salvage intratympanic corticosteroid therapy in patients with incomplete recovery after initial treatment.^158^^,^^159^^,^^161^, ^162^, ^163^, ^164^, ^165^, ^166^, ^167^, ^168^, ^169^, ^170^, ^171^, ^172^, ^173^, ^174^, ^175^, ^176^, ^177^, ^178^, ^179^, ^180^, ^181^, ^182^, ^183^

Although protocols differ significantly among studies regarding drug type, concentration, number of injections, and treatment timing, most evidence suggests that salvage intratympanic therapy provides additional hearing improvement compared with observation alone.^158^^,^^159^

Devantier et al. performed a systematic review and meta-analysis evaluating intratympanic corticosteroid therapy as salvage treatment for idiopathic SSNHL.^158^

The authors demonstrated that salvage intratympanic therapy was associated with improved hearing outcomes, although heterogeneity among studies limited definitive conclusions regarding the optimal protocol.^158^

Spear and Schwartz also reviewed the available evidence and concluded that intratympanic corticosteroids represent a reasonable therapeutic alternative for patients who do not recover after systemic corticosteroid treatment.^159^

Raymundo et al. evaluated intratympanic methylprednisolone as rescue therapy after failure of conventional treatment and reported additional hearing improvement in selected patients.^161^

Despite favorable results, variability between studies remains an important limitation. Differences include: Corticosteroid used (dexamethasone versus methylprednisolone); Drug concentration; Number and frequency of injections; Definition of hearing recovery; Time interval between onset of hearing loss and salvage therapy initiation.^158^^,^^164^^,^^165^

### Effectiveness of salvage intratympanic corticosteroid therapy

The available evidence supports the use of intratympanic corticosteroids as salvage therapy in patients with incomplete recovery after initial treatment for SSNHL.^3^^,^^58^^,^^159^

Multiple clinical studies have reported additional hearing gain after salvage intratympanic therapy, although the magnitude of benefit varies according to patient characteristics, initial hearing severity, and treatment timing.^161^, ^162^, ^163^, ^164^, ^165^, ^166^, ^167^, ^168^, ^169^, ^170^, ^171^, ^172^, ^173^, ^174^, ^175^, ^176^, ^177^, ^178^, ^179^, ^180^, ^181^, ^182^, ^183^

Several authors demonstrated that patients receiving salvage intratympanic corticosteroids achieved greater improvements in pure-tone average thresholds compared with patients who received no additional treatment after systemic corticosteroid failure.^160^^,^^169^, ^170^, ^171^, ^172^, ^173^, ^174^, ^175^, ^176^, ^177^

However, heterogeneity among studies prevents definitive conclusions regarding the superiority of a specific treatment regimen.^158^^,^^165^

### Timing of salvage treatment

The timing of intratympanic corticosteroid administration appears to be an important prognostic factor.^3^^,^^158^

Earlier initiation after failure of initial therapy has been associated with better auditory outcomes, whereas delayed salvage treatment is generally associated with reduced probability of recovery.^178^, ^179^, ^180^, ^181^, ^182^

Current guidelines recommend offering salvage intratympanic corticosteroid therapy when patients have incomplete recovery after initial management, particularly within the early therapeutic window.^3^

### Corticosteroid type and concentration

The optimal corticosteroid agent and concentration for intratympanic therapy remain uncertain.^158^^,^^164^

Dexamethasone and methylprednisolone are the most frequently studied medications.^158^^,^^164^^,^^165^

Different concentrations have been reported across studies, including variable dexamethasone protocols and high-dose methylprednisolone regimens.^164^, ^165^, ^166^, ^167^, ^168^

Pharmacokinetic studies suggest that higher concentrations may increase intracochlear exposure, although clinical superiority has not been consistently demonstrated.^160^^,^^164^^,^^165^

### Administration protocols and delivery techniques

Protocols for intratympanic corticosteroid administration vary considerably among published studies.^158^^,^^165^

Reported differences include: Number of injections; Interval between applications; Total treatment duration; Corticosteroid concentration; Delivery method.^158^^,^^164^, ^165^, ^166^, ^167^, ^168^

Most protocols use repeated transtympanic injections performed under microscopic visualization, allowing diffusion through the round window membrane.^76^^,^^164^

Alternative methods, including catheter-based continuous delivery systems and techniques designed to improve round window exposure, have also been investigated.^166^, ^167^, ^168^

Although these approaches aim to increase inner ear drug delivery, current evidence is insufficient to recommend one technique over another.^158^^,^^165^

### Factors associated with response to salvage therapy

Several factors may influence hearing recovery after salvage intratympanic corticosteroid treatment, including: Initial degree of hearing loss; Audiometric configuration; Presence of vertigo; Age; Time from symptom onset to treatment initiation.^184^, ^185^, ^186^, ^187^, ^188^, ^189^, ^190^, ^191^, ^192^, ^193^, ^194^, ^195^, ^196^, ^197^

Patients with earlier treatment initiation and less severe initial hearing loss generally demonstrate better outcomes.^186^, ^187^, ^188^, ^189^, ^190^

Nevertheless, some studies suggest that patients with severe or profound hearing loss may still benefit from salvage therapy, supporting individualized decision-making.^191^, ^192^, ^193^, ^194^, ^195^, ^196^, ^197^

## Discussion

Although corticosteroids remain the main therapeutic option for SSNHL, there is still uncertainty regarding the optimal treatment protocol, including the best route of administration, timing, dosage, and duration of therapy.^3^^,^^158^

The randomized clinical trial by Rauch et al. demonstrated that intratympanic corticosteroid therapy was not inferior to systemic corticosteroids as initial therapy for idiopathic SSNHL, supporting the biological efficacy of local corticosteroid administration.^75^

The main advantage of intratympanic therapy is the ability to achieve higher inner ear corticosteroid concentrations while avoiding systemic adverse effects.^76^^,^^160^^,^^164^

Therefore, intratympanic corticosteroids represent an especially valuable option for patients with contraindications to systemic corticosteroids, such as uncontrolled diabetes mellitus, severe hypertension, glaucoma, or other conditions associated with increased risk of systemic steroid complications.^199^^,^^200^

Despite increasing evidence supporting salvage intratympanic corticosteroid therapy, several limitations remain.

Most studies differ substantially regarding: Inclusion criteria; Definition of treatment failure; Timing of salvage therapy initiation; Corticosteroid agent and concentration; Number of applications; Criteria used to define hearing recovery.^158^^,^^159^^,^^165^

This heterogeneity limits the establishment of a universally accepted protocol.

Nevertheless, considering the possibility of permanent hearing impairment and the relatively low rate of complications associated with intratympanic injections, salvage therapy represents a reasonable option after incomplete recovery with initial treatment.^3^^,^^158^

Adverse effects associated with intratympanic corticosteroid therapy are generally mild and transient.

Reported complications include: Temporary dizziness or vertigo; Injection-related discomfort; Otalgia; Transient taste disturbance; Persistent tympanic membrane perforation (rare).^158^^,^^198^

Considering its safety profile, potential benefit, and limited therapeutic alternatives after treatment failure, intratympanic corticosteroid therapy has become an important component of SSNHL management.^3^^,^^158^

### Recommendation

Intratympanic corticosteroid therapy should be offered as salvage treatment for patients with SSNHL who present incomplete recovery after initial management.^3^^,^^158^

The greatest benefit appears to occur when treatment is initiated early after failure of systemic therapy.^3^^,^^158^^,^^184^, ^185^, ^186^, ^187^, ^188^, ^189^, ^190^, ^191^

Although there is no universally established protocol, repeated intratympanic administration of dexamethasone or methylprednisolone is currently the most frequently adopted approach.^158^^,^^164^^,^^165^

### Conclusion

Current evidence supports intratympanic corticosteroid therapy as a salvage option for patients with sudden sensorineural hearing loss who do not achieve satisfactory recovery after initial treatment.

Although variations exist among published protocols, salvage intratympanic therapy can provide additional hearing improvement with a favorable safety profile.

Future randomized controlled trials with standardized definitions of hearing recovery, treatment timing, corticosteroid concentration, and administration protocols are necessary to establish the optimal therapeutic strategy.^158^^,^^165^

## Statement 11 ‒ Hyperbaric oxygen therapy (hbot) as rescue therapy for Sudden Sensorineural Hearing Loss (SSNHL)

Clinicians may offer Hyperbaric Oxygen Therapy (HBOT), in combination with corticosteroids, as salvage therapy for patients with SSNHL and incomplete recovery after initial treatment, preferably within 2–4 weeks of symptom onset. Option.

### Action statement

Quality improvement opportunity: To evaluate and summarize the evidence for HBOT as rescue therapy in patients with SSNHL refractory to initial corticosteroid treatment, guiding clinical decision-making for this specific scenario.

Aggregate evidence quality: Grade B, based on randomized controlled trials, prospective and retrospective cohort studies, systematic reviews, and meta-analyses demonstrating benefit of HBOT when combined with corticosteroids in selected patients with refractory SSNHL.

Level of confidence in the evidence: Moderate. Evidence is consistent in identifying a subgroup of patients (severe-to-profound loss, early initiation) that benefits from salvage HBOT. Evidence for the general SSNHL population is less conclusive.

Benefits: Improved Pure-Tone Average (PTA) gains; increased rates of partial and complete auditory recovery; particularly effective in severe-to-profound loss; noninvasive; applicable when intratympanic access is unavailable or contraindicated.

Risks, harms, costs: Claustrophobia; barotrauma (otic, sinus, pulmonary); oxygen toxicity (rare at standard protocols); contraindicated in untreated pneumothorax; significant cost and logistical burden (multiple daily sessions at specialized centers); not universally available.

Benefits-harm assessment: Preponderance of benefit over harm in patients with severe-to-profound refractory SSNHL presenting within 2–4 weeks of onset, when combined with corticosteroids and initiated promptly.

Value judgments: The therapeutic window is critical: HBOT initiated beyond 3–4 weeks from symptom onset has not demonstrated benefit. Clinical urgency in defining treatment failure and initiating rescue therapy is essential

Intentional vagueness: Optimal number of sessions, exact pressure protocol, and upper time limit for rescue initiation remain incompletely defined; current best evidence supports 2.5 ATA / 60-min / 10 sessions as the minimum effective protocol

Role of patient preferences: Moderate. Patient willingness to commit to multiple sessions and tolerance of chamber confinement are relevant. Shared decision-making is important given equivalence with intratympanic steroids in comparative studies

Exceptions: Untreated pneumothorax; severe claustrophobia; certain chemotherapy agents (bleomycin, doxorubicin); active middle ear or sinus infection; SSNHL onset > 4 weeks prior (insufficient evidence of benefit)

Policy level: Option ‒ HBOT combined with corticosteroids may be offered as rescue therapy for patients with SSNHL refractory to initial treatment, preferably within 2–4 weeks of onset.

Differences of opinion: Minor disagreement existed regarding whether HBOT should be classified as a recommendation or an option because of resource utilization, availability, and variability in treatment protocols. However, consensus was reached that HBOT represents a valid salvage treatment when combined with corticosteroids and initiated within the appropriate therapeutic window.

**Supporting text**

A significant proportion of patients with SSNHL treated with systemic corticosteroids (estimated between 30% and 50%) fails to recover adequately with initial therapy, defining refractory SSNHL (RSSNHL).

For these patients, rescue (salvage) therapy is required. The most studied options include intratympanic corticosteroids (ITS), Hyperbaric Oxygen Therapy (HBOT), and, more recently, their combination.

HBOT consists of the inhalation of 100% oxygen at pressures greater than atmospheric (typically 2.0–2.5 absolute atmospheres, ATA), producing systemic hyperoxia.

Within the cochlea, this substantially elevates perilymph oxygen tension, potentially reversing the hypoxic injury that underlies SSNHL in vascular-mechanism cases.

It was first employed for SSNHL in the late 1970s^152^ and was formally recognized as an accepted clinical indication by the Undersea and Hyperbaric Medical Society (UHMS) in 2011.^201^

The 2019 AAO-HNS Clinical Practice Guideline Update recommends HBOT as an option (Grade B) exclusively in combination with corticosteroids ‒ both as initial and as rescue therapy ‒ preferably within 4-weeks of symptom onset.^3^

## Methods

We conducted a systematic review following PRISMA 2020 guidelines^228^ to evaluate the efficacy of HBOT as rescue therapy in patients with SSNHL refractory to initial treatment.

The databases searched were PubMed/MEDLINE, Embase, Cochrane Library (CENTRAL), Scopus, and Web of Science, from inception to April 2025, with no language restrictions.

The search strategy combined three blocks using Boolean operators:

• Block 1 (Condition): ("sudden hearing loss"[MeSH] OR "sudden sensorineural hearing loss"[tiab] OR "SSNHL"[tiab] OR "sudden deafness"[tiab])
• Block 2 (Intervention): ("hyperbaric oxygenation"[MeSH] OR "hyperbaric oxygen therapy"[tiab] OR "HBOT"[tiab] OR "hyperbaric oxygen"[tiab])
• Block 3 (Rescue context): ("rescue therapy"[tiab] OR "salvage therapy"[tiab] OR "refractory"[tiab] OR "treatment failure"[tiab])

Approximately 768 records were identified across all databases.

After removal of approximately 308 duplicates, 460 unique records were screened by title and abstract.

Following full-text review of 106 articles, 30 studies were included in the qualitative synthesis: 17 primary studies; 8 systematic reviews and meta-analyses; 3 normative documents (guidelines and health technology assessments).

### Research question (PICO format)

P – Population: Adults (≥ 18-years) with idiopathic unilateral SSNHL who failed initial corticosteroid therapy (PTA gain < 10 dB)

I – Intervention: HBOT as rescue therapy, alone or combined with intratympanic or systemic corticosteroids.

C – Comparison: No additional treatment, intratympanic steroids (ITS) alone, continued systemic steroids, or other rescue modalities.

O – Outcomes:

Primary: Mean PTA gain (dB); Complete/partial recovery rate (Siegel or Furuhashi criteria).

Secondary: Time to recovery; Frequency-specific response; Prognostic factors.

### Methodological quality

Randomized controlled trials were assessed with the Cochrane Risk of Bias tool (RoB2.0).

All three RCTs identified were rated as having “some concerns” in the domain of deviations from intended interventions, due to the structural impossibility of blinding participants and outcome assessors to HBOT.

Non-randomized studies were assessed with the Newcastle-Ottawa Scale (NOS).

Most retrospective and prospective non-randomized studies scored 5–7 out of 9 stars, reflecting moderate methodological quality.

The main limitations were: Absence of formal randomization; Selection bias; Variability in follow-up duration; Heterogeneity in outcome reporting.

The two highest-quality primary studies ‒ Lee et al.^202^ and Cvorovic et al.^219^ ‒ both scored 7/9 stars on the NOS, with contemporary control groups and clearly defined failure criteria.

### Effectiveness of HBOT as salvage therapy

The available evidence suggests that HBOT may provide additional hearing recovery in patients with refractory SSNHL, particularly when combined with corticosteroid therapy.^202^, ^203^, ^204^, ^205^, ^206^, ^207^, ^208^, ^209^, ^210^

However, the magnitude of benefit varies considerably among studies due to differences in patient selection, baseline hearing severity, timing of treatment initiation, and HBOT protocols.^203^, ^204^, ^205^, ^206^

Lee et al. evaluated HBOT as salvage therapy in patients with refractory SSNHL and demonstrated significant hearing improvement compared with patients who did not receive additional treatment.^202^

The benefit was more pronounced among patients with severe-to-profound hearing loss, suggesting that baseline severity may influence the magnitude of therapeutic response.^202^^,^^211^

Several systematic reviews and meta-analyses have investigated the role of HBOT in SSNHL.^203^, ^204^, ^205^, ^206^, ^207^, ^208^, ^209^, ^210^

Joshua et al. demonstrated that HBOT combined with medical therapy was associated with significantly higher hearing improvement compared with medical therapy alone.^203^

Similarly, Eryigit et al. reported favorable outcomes with HBOT, although they emphasized heterogeneity among included studies and the need for better-designed randomized trials.^210^

The Cochrane review evaluating HBOT for idiopathic sudden sensorineural hearing loss concluded that HBOT may improve hearing outcomes, especially when administered early, but highlighted limitations related to study quality and variability in protocols.^216^

Recent umbrella reviews have reinforced these conclusions, suggesting a possible benefit while emphasizing that certainty of evidence remains limited.^218^

### HBOT versus intratympanic corticosteroids as salvage therapy

Several studies compared HBOT with intratympanic corticosteroids (ITS) as rescue treatment for refractory SSNHL.^204^, ^205^, ^206^^,^^220^, ^221^, ^222^, ^223^, ^224^, ^225^, ^226^, ^227^

Current evidence does not demonstrate consistent superiority of one salvage modality over the other.

Lei et al. compared HBOT and ITS through meta-analysis and reported comparable hearing outcomes between the two interventions.^204^

Kuo et al. also found no definitive superiority, suggesting that patient characteristics and treatment availability should guide therapeutic selection.^205^

Combination therapy (HBOT + corticosteroids) has been proposed to maximize potential benefit by targeting different mechanisms:

• HBOT → improves cochlear oxygenation and reduces hypoxia-related injury;
• Corticosteroids → reduce inflammation and immune-mediated cochlear damage.^203^^,^^224^

Yang et al. compared intratympanic steroid injection, HBOT, and combined treatment strategies and suggested that combined therapy may provide additional benefit in selected patients.^224^

Other comparative studies have reported similar findings, particularly among patients with severe or profound hearing loss.^221^, ^222^, ^223^, ^224^, ^225^, ^226^

### Influence of timing on treatment response

Time from symptom onset to HBOT initiation appears to be one of the most important prognostic factors.^3^^,^^203^^,^^217^

Earlier initiation of HBOT is consistently associated with better auditory recovery.^203^^,^^208^^,^^217^

Delayed treatment may reduce therapeutic effectiveness due to irreversible cochlear damage and loss of viable sensory cells.^203^

Current recommendations suggest that HBOT should preferably be performed within the early treatment window, particularly within 4-weeks of symptom onset when used as salvage therapy.^3^

### Influence of initial hearing severity

Patients with severe or profound SSNHL appear to derive the greatest benefit from HBOT.^211^^,^^212^

Choi et al. demonstrated improved outcomes in severe-to-profound cases treated with HBOT, supporting the hypothesis that patients with greater cochlear injury may experience larger measurable gains.^212^

Kim et al. also reported that optimized HBOT protocols may improve outcomes, especially in patients with more severe baseline hearing thresholds.^212^

### HBOT protocol considerations

HBOT protocols vary substantially among published studies.^203^^,^^214^

Commonly used regimens include: Pressure between 2.0 and 2.5 ATA; Session duration of approximately90-minutes; Daily treatments; Total number of sessions ranging from 10 to 20.^201^^,^^214^

Filipo et al. evaluated modifications in HBOT schedules and suggested that treatment intensity may influence outcomes.^213^

However, there is currently insufficient evidence to define an optimal pressure, duration, or number of sessions.^203^^,^^216^

### Mechanisms supporting HBOT use in refractory SSNHL

The therapeutic rationale for HBOT in refractory SSNHL is based primarily on the hypothesis that cochlear hypoxia contributes to auditory dysfunction.^152^^,^^201^

The cochlea has high metabolic demand and limited vascular supply, making it particularly vulnerable to alterations in oxygen delivery.

HBOT increases dissolved oxygen concentration in plasma and enhances oxygen diffusion into inner ear fluids, especially the perilymph, potentially improving oxygen availability to injured sensory and neural structures.^152^^,^^201^

In addition to increasing oxygen tension, HBOT may exert additional biological effects, including: Reduction of cochlear edema; Improvement of microcirculation; Modulation of inflammatory responses; Reduction of ischemia-related injury.^201^^,^^214^

These mechanisms provide the theoretical basis for combining HBOT with corticosteroids, since both therapies may act through complementary pathways.^3^^,^^203^

### Safety and adverse effects

HBOT is generally considered a safe intervention when performed under appropriate protocols.^201^^,^^214^

However, potential adverse effects should be considered, including: Middle ear barotrauma; Ear discomfort or pain; Sinus barotrauma; Temporary visual changes; Claustrophobia; Rare oxygen toxicity events.^201^^,^^214^

The need for repeated sessions, specialized equipment, and trained personnel represents an important limitation regarding accessibility and feasibility.^214^^,^^215^

### Limitations of current evidence

Despite multiple studies suggesting potential benefit, important limitations remain in the available literature.^203^^,^^216^^,^^218^

The main limitations include: Limited number of randomized controlled trials; Small sample sizes; Heterogeneous inclusion criteria; Differences in baseline hearing severity; Variable timing of HBOT initiation; Different HBOT protocols; Lack of standardized definitions of hearing recovery.^203^, ^204^, ^205^, ^206^^,^^216^

Health technology assessments have emphasized that these methodological limitations reduce certainty regarding the magnitude of treatment effect and cost-effectiveness.^215^

### Clinical interpretation

Available evidence suggests that HBOT should not be considered a universal salvage therapy for all patients with refractory SSNHL.^3^^,^^215^

However, selected patients may benefit, particularly: Severe-to-profound SSNHL; Recent onset of hearing loss; Incomplete recovery after corticosteroid therapy; Patients in whom additional hearing recovery would have significant functional impact.^3^^,^^202^^,^^211^

The decision to offer HBOT should consider: Expected benefit; Treatment availability; Cost; Patient preference; Associated comorbidities.^214^^,^^215^

### Recommendation

HBOT may be considered as salvage therapy in selected patients with SSNHL who present incomplete recovery after initial corticosteroid treatment, especially in cases of severe-to-profound hearing loss and when therapy can be initiated within the recommended therapeutic window.^3^^,^^202^^,^^211^

Current evidence does not support routine use of HBOT as salvage treatment for all patients due to limited availability, cost, and uncertainty regarding benefit magnitude.^215^^,^^216^

### Conclusion

Hyperbaric oxygen therapy represents a biologically plausible salvage treatment option for refractory sudden sensorineural hearing loss.

Evidence suggests potential additional hearing recovery, particularly when HBOT is combined with corticosteroids and used early in patients with severe or profound hearing loss.^203^^,^^211^^,^^217^

However, heterogeneity among studies and limited high-quality randomized evidence prevent definitive recommendations regarding universal use.

Future studies should focus on standardized protocols, patient selection criteria, cost-effectiveness analysis, and identification of subgroups most likely to benefit from HBOT.^206^^,^^218^

## Statement 12 ‒ Antivirals, thrombolytics, vasodilators, and vasoactive substances

Clinicians should not routinely prescribe antivirals, thrombolytics, vasodilators, vasoactive substances, or related agents for the treatment of idiopathic sudden sensorineural hearing loss. Recommendation against routine use.

### Action statement

Quality improvement opportunity: Reduce the use of ineffective or insufficiently validated therapies in patients with idiopathic SSNHL, promote evidence-based treatment strategies, avoid unnecessary adverse effects and healthcare expenditures, and improve adherence to established clinical practice guidelines.

Aggregate evidence quality: Grade B, based on randomized controlled trials, clinical practice guidelines, systematic reviews, and prospective studies consistently demonstrating insufficient evidence to support routine use of antivirals, thrombolytics, vasodilators, vasoactive substances, or related agents in idiopathic SSNHL.

Level of confidence in the evidence: High.

Benefits: Avoid unnecessary medication exposure; reduce treatment-related adverse effects; decrease healthcare costs; prevent delays in initiating evidence-based therapies; reduce polypharmacy; improve adherence to guideline-directed management.

Risks, harms, costs: Potential failure to identify a small subgroup of patients who could theoretically benefit from mechanism-specific therapies; adverse drug reactions; bleeding risk associated with thrombolytic therapies; cardiovascular adverse effects associated with vasoactive agents; drug interactions; increased treatment costs without demonstrated clinical benefit.

Benefits-harm assessment: Preponderance of benefit over harm for avoiding routine use.

Value judgments: The guideline panel recognized the biological plausibility of viral, vascular, thrombotic, and microcirculatory mechanisms in the pathogenesis of SSNHL. However, despite these theoretical considerations, available clinical studies have not consistently demonstrated meaningful improvement in hearing outcomes with routine use of antivirals, thrombolytics, vasodilators, vasoactive agents, or related therapies. The panel placed greater value on evidence-based interventions with demonstrated clinical benefit.

Intentional vagueness: The statement does not address highly selected patients in whom treatment may be directed toward a specific identified etiology. Future advances in patient phenotyping, biomarker identification, and precision medicine approaches may allow more individualized therapeutic strategies.

Role of patient preferences: Limited. Patients may express interest in additional therapies; however, clinicians should counsel patients regarding the lack of consistent evidence supporting routine use of these agents in idiopathic SSNHL.

Exceptions: Patients with an identified underlying condition for which antiviral, antithrombotic, vasoactive, or other targeted therapies are independently indicated based on the specific diagnosis rather than the diagnosis of idiopathic SSNHL itself.

Policy level: Recommendation against routine use.

Differences of opinion: Minor disagreement existed regarding the possible role of selected therapies in severe-to-profound hearing loss or in specific pathophysiological subgroups. However, consensus was reached that current evidence remains insufficient to support routine prescription of these agents in patients with idiopathic SSNHL.

**Supporting text**

The routine use of antivirals, thrombolytics, vasodilators, or vasoactive substances has been discouraged in previous Clinical Practice Guidelines (CPG).^3^^,^^89^

The purpose of our review is to update the current evidence regarding the efficacy of these alternative therapies in the context of Idiopathic Sudden Sensorineural Hearing Loss (ISSNHL).

## Methods

We conducted a Systematic Review (SR) in accordance with the guidelines of the Preferred Reporting Items for Systematic Reviews and Meta-Analyses (PRISMA) statement.^228^

The research question applied was: “Are antivirals, thrombolytics, vasodilators, or vasoactive substances effective in improving hearing function in idiopathic sudden sensorineural hearing loss?”

A systematic search of the literature was performed in key databases: MEDLINE; EMBASE; Web of Science; Cumulative Index to Nursing and Allied Health Literature; Cochrane Database of Systematic Reviews.

The search strategy used in all databases was: (“Hearing Loss, Sudden”[Mesh] OR “sudden hearing loss” OR “sudden deafness” OR “sudden sensorineural hearing loss” OR “idiopathic sudden hearing loss”) AND ("Antiviral Agents"[Mesh] OR "Thrombolytic Therapy"[Mesh] OR "Fibrinolytic Agents"[Mesh] OR "Vasodilator Agents"[Mesh] OR “Vasoactive”)

All databases were last searched on July 15th, 2025.

We used Zotero and Rayyan softwares to increase the reliability of article selection and identification of duplicate studies before the eligibility stage.

Two independent reviewers, blinded to each other's decisions, reviewed the studies. Any disagreements were solved through discussion.

### Eligibility criteria

The inclusion criteria were:

1 Studies including only patients with ISSNHL ‒ sensorineural hearing loss of at least 30 dB in three consecutive frequencies occurring within a 72 -h window;
2 Studies in which antivirals, thrombolytics, vasodilators, or vasoactive substances were used in the intervention group;
3 Studies in which the outcome criterion was defined as hearing improvement according to Pure Tone Average (PTA);
4 Clinical practice guidelines, systematic reviews, prospective trials, and observational studies.

The exclusion criteria were: Non-English papers; Studies published before 2015; Incomplete studies or unpublished clinical trials; Animal or in-vitro studies; Case reports and retrospective studies; Patients younger than 18-years-old; Patients with an identifiable etiology for sudden sensorineural hearing loss; Inadequate reporting of data.

We initially planned to include exclusively placebo-controlled studies.

Nevertheless, there is still no strong evidence to recommend the use of the drugs included in this review as monotherapy or to compare them only with placebo.

Therefore, studies comparing these treatments in association with other therapies were also included.

We also initially planned to include only studies defining hearing gain as improvement of at least 10 dB in PTA after treatment.

However, there is considerable heterogeneity among studies regarding hearing thresholds and outcome criteria.

Some studies consider effective hearing gain as an improvement in PTA of at least 10 or 15 dB, or as a percentage of improvement compared with the contralateral ear threshold.

ISSNHL research remains limited by lack of standardization of outcome measures.^229^

### Study selection

The systematic search identified 386 studies.

A total of 28 duplicates were removed.

After title and abstract analysis, 16 studies remained.

During full-text analysis, 7 studies were excluded due to: Inappropriate study design (retrospective)^230^, ^231^, ^232^, ^233^; Ineligible population (patients younger than 18-years old or non-idiopathic cases);^234^^,^^235^ Inadequate reporting of hearing data.^236^

A total of 9 studies were included in the qualitative synthesis.

### Pathophysiological rationale and alternative therapies

Sudden sensorineural hearing loss, also referred to as sudden deafness, is a clinical condition characterized by a rapid loss of hearing rather than a specific disease entity.

It may result from a wide variety of etiologies or its cause may be multifactorial.^229^

Several theories have been proposed regarding its origin, including viral infection^237^, ^238^, ^239^ and blood disorders in microcirculation causing cochlear ischemia.^240^

### Antiviral therapy

Antiviral efficacy in ISSNHL was assessed in a non-randomized trial through the comparison of other drugs in association or not with famciclovir.^241^

No statistically significant difference was found between groups regarding hearing improvement.^241^

Therefore, current evidence does not support routine antiviral therapy for patients with ISSNHL.^3^^,^^89^

### Prostaglandin E1 and vasodilator therapy

Prostaglandin E1 (PGE1) is an arachidonic acid metabolite with vasodilatory properties, empirically used with the objective of increasing cochlear blood flow and oxygen supply.

Xi et al. evaluated the effects of PGE1 in a randomized controlled trial comparing the combination of prednisolone and ginkgo leaf extract, associated or not with PGE1.^242^

The difference between groups was not statistically significant.^242^

Kitoh et al., in the clinical practice guideline developed by the Research Group of the Ministry of Health, Labour and Welfare (MHLW) Japan, evaluated PGE1 compared with placebo, both combined with hydrocortisone.^148^

They reported that PGE1 may be an option in severe-to-profound cases, although there is no clear evidence to support its efficacy.^148^

Feng et al. evaluated alprostadil, a synthetic form of PGE1, as monotherapy compared with the combination of alprostadil and hyperbaric oxygen therapy.^243^

The combination group showed a higher effective treatment rate; however, symptom relief and hearing improvement were analyzed simultaneously, limiting interpretation of isolated hearing outcomes.^243^

### Pentoxifylline

Pentoxifylline is a hemorheologic agent that modifies blood viscosity, theoretically improving cochlear microcirculation.

A systematic review by Ramzi et al. found insufficient evidence to recommend pentoxifylline as monotherapy for inner ear diseases.^244^

Conflicting results were reported regarding whether pentoxifylline combined with systemic corticosteroids could achieve similar efficacy compared with corticosteroid monotherapy.^244^

### Therapeutic apheresis

Therapeutic apheresis is the selective removal of pathogenic substances from the patient's blood using an extracorporeal medical device.^245^

The techniques evaluated for ISSNHL include: HELP-apheresis; Rheopheresis.

These techniques differ according to which plasma components are filtered during the procedure.

The objective is to reduce lipoprotein levels, improve hemorheology, and increase cochlear blood flow.^247^, ^248^, ^249^

Moreno-Herraiz et al. evaluated the efficacy of apheresis for sudden sensorineural hearing loss in a systematic review and meta-analysis.^246^

Apheresis demonstrated improvement in hearing function.^246^

However, important limitations were identified, including: Heterogeneity of study designs; Differences in patient characteristics; Variation in the number of apheresis sessions; Geographic concentration of available evidence, with most studies originating from Germany.^246^

Therefore, despite promising results, current evidence remains insufficient to recommend routine therapeutic apheresis for all patients with ISSNHL.^246^

### Defibrinogenation therapy and thrombolytic agents

Based on the hypothesis that alterations in cochlear microcirculation may contribute to sudden sensorineural hearing loss, therapies targeting blood viscosity and coagulation pathways have been investigated.^240^

Defibrinogenation therapy aims to reduce fibrinogen concentration, decrease blood viscosity, and improve cochlear perfusion.^250^

Batroxobin, a thrombin-like serine protease extracted from snake venom, has been evaluated as a fibrinogen-lowering agent in ISSNHL.

Oya et al. investigated the efficacy of batroxobin treatment in patients with sudden sensorineural hearing loss.^251^

Although some studies reported potential hearing improvement, the available evidence remains limited by heterogeneity in study design, patient selection, and associated treatments.^251^

Therefore, current data are insufficient to support routine use of thrombolytic or defibrinogenation therapy in ISSNHL.^3^^,^^89^

### Zinc supplementation

Zinc has been investigated as a potential therapeutic agent due to its biological role in cochlear function.

Experimental studies suggest that zinc participates in auditory neurotransmission and may influence neuronal survival mechanisms.^252^

Additionally, antioxidant pathways involving copper/zinc superoxide dismutase (Cu/Zn-SOD) have been identified in cochlear structures, suggesting a possible protective effect against oxidative stress.^253^

Early clinical studies proposed a potential association between zinc deficiency and auditory disorders, including presbycusis and tinnitus.^254^^,^^255^

Experimental evidence also suggests that zinc may influence cochlear protection mechanisms.^256^^,^^257^

Hunchaisri et al. evaluated zinc supplementation as an adjunctive treatment in idiopathic sudden sensorineural hearing loss.^258^

Although some improvement was reported, available evidence remains insufficient to establish zinc as a standard therapeutic option for ISSNHL.^258^

### Clinical interpretation

Despite multiple proposed mechanisms, including viral infection, cochlear ischemia, microvascular dysfunction, oxidative stress, and inflammatory pathways, there is currently insufficient evidence to recommend routine use of: Antivirals; Thrombolytics; Vasodilators; Vasoactive substances; Zinc supplementation; for patients with idiopathic sudden sensorineural hearing loss.^3^^,^^89^

Most available studies present important methodological limitations, including: Small sample sizes; Lack of placebo-controlled designs; Heterogeneous treatment protocols; Combined therapies preventing evaluation of isolated effects; Variable definitions of hearing recovery.^229^

### Recommendation

Antivirals, thrombolytics, vasodilators, and vasoactive substances should not be routinely prescribed for patients with idiopathic sudden sensorineural hearing loss due to insufficient evidence demonstrating consistent clinical benefit.^3^^,^^89^

Their use should be restricted to specific clinical scenarios, research protocols, or selected patients with individualized indications.

### Conclusion

Current evidence does not support the routine use of antiviral agents, thrombolytics, vasodilators, or vasoactive substances as standard therapy for ISSNHL.

Although several biological mechanisms have been proposed, including viral and vascular hypotheses, clinical studies have failed to demonstrate consistent improvement in hearing outcomes.^237^, ^238^, ^239^, ^240^

Future randomized controlled trials with standardized diagnostic criteria, treatment protocols, and outcome measures are required to clarify whether specific patient subgroups may benefit from these therapies.^229^

## Statement 13 ‒ Other pharmacological therapies – immunosuppressors and immunobiological agents

Clinicians should not routinely prescribe immunosuppressive agents, immunobiological therapies, neurotrophic factors, stem cell therapies, or other biological agents for the treatment of SSNHL outside approved clinical research protocols. Recommendation against routine use.

### Action statement

Quality improvement opportunity: Prevent the use of unproven and potentially costly therapies in patients with SSNHL, promote evidence-based treatment, encourage enrollment in well-designed clinical trials, and avoid exposing patients to interventions with uncertain efficacy and safety.

Aggregate evidence quality: Grade C, based on limited randomized controlled trials, early-phase clinical studies, preclinical investigations, and heterogeneous evidence demonstrating insufficient clinical efficacy to support routine use of immunosuppressive, immunobiological, regenerative, or targeted biological therapies in SSNHL.

Level of confidence in the evidence: Moderate.

Benefits: Avoid unnecessary exposure to therapies with uncertain clinical benefit; reduce treatment-related costs; minimize risks associated with immunosuppression and experimental interventions; encourage use of established therapies with demonstrated efficacy; promote participation in controlled clinical research.

Risks, harms, costs: Potential adverse effects of immunosuppressive and biologic agents; increased healthcare costs; identification of treatment-related complications without proven clinical benefit; false expectations regarding efficacy; limited availability of specialized therapies; uncertainty regarding long-term safety.

Benefits-harm assessment: Preponderance of benefit over harm for avoiding routine clinical use outside research settings.

Value judgments: The guideline panel acknowledged the strong biological rationale supporting immune modulation, neuroprotection, and regenerative approaches in SSNHL. However, despite encouraging preclinical findings and isolated positive clinical studies, the available evidence remains insufficient, inconsistent, and largely exploratory. The panel placed greater value on therapies supported by established clinical evidence while encouraging continued investigation of novel biological treatments.

Intentional vagueness: The statement does not exclude future incorporation of specific biological therapies into clinical practice should adequately powered randomized controlled trials demonstrate efficacy and safety. Emerging therapies and biomarkers may eventually allow more personalized treatment approaches.

Role of patient preferences: Limited. Patients may express interest in innovative or experimental therapies; however, clinicians should provide clear counseling regarding the investigational nature of these interventions and the absence of sufficient evidence supporting routine clinical use.

Exceptions: Participation in approved clinical trials; patients treated within formal research protocols; selected cases managed in highly specialized centers under institutional review and regulatory oversight.

Policy level: Recommendation against routine use.

Differences of opinion: Minor disagreement existed regarding the potential future role of selected biological therapies, particularly intratympanic IGF-1 and targeted immunomodulatory agents. However, consensus was reached that current evidence remains insufficient to support routine clinical use of these interventions outside research settings.

**Supporting text**

## Introduction

In recent years, increasing attention has turned toward biological agents, which are therapeutics derived from or targeting biological processes, as potential interventions for SSNHL.^262^, ^263^, ^264^

These include neurotrophic factors (such as BDNF and IGF-1), anti-inflammatory cytokine inhibitors (e.g., anti-TNF agents), and Mesenchymal Stem Cells (MSCs) capable of promoting regeneration and modulating immune responses within the inner ear.^262^

Preclinical studies and early-phase clinical trials have suggested these agents may support neuronal survival, synaptic repair, and vascular stabilization in the cochlea.^262^^,^^264^

This systematic review aims to critically evaluate the current evidence supporting the use of biological agents in the management of SSNHL, including their mechanisms of action, routes of administration, safety profiles, and therapeutic outcomes, to better inform future clinical applications and research priorities.

## Methods

This systematic review was registered in the International Prospective Register of Systematic Reviews (PROSPERO; registration number CRD420251086642).

Informed consent was waived because the data were available and legally accessible to the public and appropriately protected by law.

This study followed the Preferred Reporting Items for Systematic Reviews and Meta-Analyses (PRISMA) reporting guideline.

### Search strategy

PubMed/MEDLINE, clinical trial registry (ClinicalTrials.gov), Google Scholar, and Web of Science databases were searched using MeSH terms: “hearing loss, sudden” OR “sudden hearing loss” OR “sudden deafness” OR “sudden sensorineural hearing loss” OR “idiopathic sudden hearing loss” AND “immunotherapy” OR “immunobiology”.

The reference lists of selected articles were also searched for additional articles.

### Study selection

We included prospective randomized controlled trials involving adult participants (> 18-years old), published in English until July 2025, using the diagnostic criteria outlined by the American Academy of Otolaryngology-Head and Neck Surgery.^3^

The search was restricted to comparable immunosuppressors and immunobiological therapies as single therapy or as part of combination treatment protocols with other treatments, including oral steroids, intratympanic steroids, intravenous steroids, or placebo.

### Data extraction

Two investigators independently screened the articles and extracted data from the included studies using a standardized extraction form.

For each study, the following variables were recorded: Year of publication; Authors; Study design; Sample size; Participants’ age and sex distribution; Severity of hearing loss; Immunosuppressive or immunobiological therapy used; Pure-tone average thresholds before and after treatment.

### Grade of evidence definitions

Two researchers evaluated the grade of evidence in the included studies.

The assessment covered five domains: A ‒ Strong recommendation, high-quality evidence; B ‒ Moderate recommendation, moderate-quality evidence; C ‒ Weak recommendation, low-quality or inconsistent evidence; D ‒ Recommendation against use; I ‒ Insufficient evidence to recommend for or against use.

## Results

### Search yield

The initial search of scientific databases yielded 50 records.

An additional 25 articles were identified through gray literature.

After screening 75 articles, 52 were excluded for not meeting inclusion criteria.

Ultimately, 23 articles were included in this review.

### Anti-TNF agents (etanercept, infliximab, adalimumab)

Tumor Necrosis Factor-alpha (TNF-α) is a key pro-inflammatory cytokine hypothesized to play a role in the pathogenesis of cochlear injury in SSNHL.^265^

This hypothesis derives from the observation of elevated TNF-α levels in cochlear fluids and systemic circulation following acute inner ear insult, where it promotes inflammation, induces apoptosis of sensory hair cells and spiral ganglion neurons, and disrupts cochlear microcirculation.^265^^,^^266^

These effects may exacerbate cochlear damage and contribute to hearing loss.

Animal models of immune-mediated hearing loss suggest protective effects of TNF-α blockade on cochlear structure and auditory thresholds.^266^^,^^267^

Human data remain limited to case series and anecdotal use, mostly in autoimmune inner ear disease rather than idiopathic SSNHL.^266^

*Evidence level*: I

### Mycophenolate mofetil

Mycophenolate mofetil has been widely used as an immunosuppressive agent in organ transplantation.^262^

It blocks a wide range of cytokines, including TNF-α, interleukins 1α and 1β, and interferon-γ.^262^

Insufficient data are available in the literature to determine whether mycophenolate mofetil is effective for the treatment of SSNHL.

*Evidence level*: I

### Methotrexate

Methotrexate acts by inhibiting dihydrofolate reductase, an enzyme required for folate-dependent synthesis of purines and thymidylate, thereby blocking DNA replication and cell proliferation, particularly in rapidly dividing cells.^268^

It inhibits several pro-inflammatory pathways and cytokines, including the receptor activator of nuclear factor-kB ligand pathway, TNF, and IL-1, while increasing expression of the regulatory cytokine IL-10.^262^

No evidence currently supports the use of methotrexate for treatment of sudden sensorineural hearing loss.

Studies in patients with autoimmune inner ear disease demonstrated that methotrexate was not superior to placebo in maintaining hearing improvement achieved after prednisone therapy.^269^

*Evidence level*: D

### Neurotrophic Factors (BDNF, IGF-1, NGF)

Neurotrophins, such as Brain-Derived Neurotrophic Factor (BDNF) and Insulin-like Growth Factor-1 (IGF-1), have demonstrated efficacy in preclinical models by promoting spiral ganglion neuron survival and synaptic repair.^270^^,^^271^

A randomized controlled clinical trial involving patients with sudden sensorineural hearing loss refractory to systemic corticosteroids demonstrated that patients who underwent intratympanic IGF-1 treatment presented better recovery of hearing thresholds compared with patients treated with intratympanic dexamethasone.^272^

However, no further studies have expanded or replicated these findings, and recent evidence remains largely based on preclinical data.

*Evidence level*: I

### Mesenchymal Stem Cells (MSCs)

Mesenchymal stem cells have demonstrated the ability to modulate cochlear inflammation, promote angiogenesis, and enhance tissue regeneration in animal models.^273^

Delivery methods investigated include systemic infusion and transtympanic administration.^273^

Currently, most studies involving MSCs focus on experimental models of sensorineural hearing loss induced by: Noise trauma; Ototoxic drugs; Direct cochlear injury; rather than classical idiopathic sudden sensorineural hearing loss.^274^^,^^275^

Preclinical data suggest that MSCs may: Modulate inflammatory responses; Secrete protective factors; Promote repair mechanisms; Improve auditory thresholds in cochlear injury models.^273^^,^^275^

These findings support a biologically plausible therapeutic role for MSCs in SSNHL.

However, no large, well-controlled clinical trials have directly evaluated MSC therapy in patients with idiopathic SSNHL.

Therefore, its use remains investigational and restricted to experimental or research settings.

*Evidence level*: I

### c-Jun N-terminal kinase antagonists (AM-111 / Brimapitide)

AM-111 is a c-Jun N-terminal Kinase (JNK) pathway inhibitor delivered through intratympanic administration.^276^^,^^277^

JNK signaling is activated in response to cochlear stress and is involved in hair cell apoptosis.^276^^,^^277^

By blocking this pathway, AM-111 aims to prevent irreversible sensory hair cell damage and preserve hearing.^276^

Although preclinical studies demonstrated significant potential for AM-111 in the treatment of sudden sensorineural hearing loss, clinical results have been inconsistent.

Two phase III randomized, double-blind, placebo-controlled trials failed to demonstrate significant benefit compared with placebo in the overall SSNHL population.^276^^,^^277^

One trial did not demonstrate superiority over placebo in the global cohort, while another failed to meet the primary endpoint of improved hearing recovery at day 28.^276^^,^^277^

Although both trials suggested a possible benefit in the subgroup of patients with profound hearing loss at baseline, these findings were derived from post-hoc analyses and require further validation before clinical application.

*Evidence level*: I

## Discussion

Sudden Sensorineural Hearing Loss (SSNHL), first described in 1944, remains largely idiopathic, with unclear pathophysiology.^259^

Immune-mediated mechanisms are suspected in a subset of cases, where circulating antibodies or activated T-cells target inner ear antigens, potentially triggered by viral infections, leading to cochlear damage.^262^^,^^266^

Evidence supporting immune involvement includes the presence of autoantibodies (such as anti-heat shock protein 70 and antiphospholipid antibodies), altered immune cell profiles, inflammatory pathway activation, oxidative stress, and complement activation, which may contribute to vascular injury and sudden hearing loss.^260^^,^^261^^,^^262^^,^^266^

Despite this, the precise role of immune dysfunction and the therapeutic potential of biologics in SSNHL are still under investigation.

In this guideline, we systematically reviewed the available evidence on biological agents for Sudden Sensorineural Hearing Loss (SSNHL), including: Anti-TNF agents; Immunosuppressants; Neurotrophic factors; Mesenchymal stem cells (MSCs); JNK pathway inhibitors such as AM-111.

Despite promising biological rationale and encouraging preclinical data for many of these therapies, the current clinical evidence remains insufficient to support their routine use in the management of SSNHL.

Anti-TNF agents, although supported by preclinical studies demonstrating reduced cochlear inflammation and preservation of auditory function, have limited evidence in humans, predominantly in autoimmune inner ear disease rather than idiopathic SSNHL.^262^^,^^264^, ^265^, ^266^, ^267^

Similarly, immunosuppressive agents such as mycophenolate mofetil and methotrexate lack robust clinical trial evidence in SSNHL.

Methotrexate failed to demonstrate superiority over placebo in maintaining hearing improvement in patients with autoimmune inner ear disease.^268^^,^^269^

Among neurotrophic factors, IGF-1 represents the therapy with the strongest clinical evidence.

A randomized controlled trial demonstrated improved hearing recovery with intratympanic IGF-1 in steroid-refractory SSNHL compared with intratympanic dexamethasone.^272^

However, this finding has not been replicated or expanded in subsequent studies, limiting its incorporation into routine clinical practice.

Mesenchymal stem cells represent a promising regenerative and immunomodulatory approach.

Multiple animal studies have demonstrated potential benefits regarding cochlear repair, inflammatory modulation, and functional recovery.^273^, ^274^, ^275^

Nevertheless, no large or well-controlled clinical trials have directly evaluated MSC therapy in patients with idiopathic SSNHL.

Therefore, clinical applicability remains uncertain.^273^^,^^274^

AM-111, a JNK pathway inhibitor, demonstrated promising results in preclinical models by preventing cochlear hair cell apoptosis.^276^^,^^277^

However, two phase III randomized clinical trials failed to demonstrate significant benefit over placebo in the overall SSNHL population.^276^^,^^277^

These findings demonstrate a substantial gap between biological plausibility and proven clinical efficacy for biological agents in SSNHL.

Current evidence remains insufficient to justify routine clinical use outside controlled research settings.

There is a need for well-designed, adequately powered randomized clinical trials to assess: Safety; Efficacy; Appropriate patient selection; Treatment timing; Optimal delivery methods.

Until sufficient evidence supporting biological agents becomes available, corticosteroids remain the mainstay of SSNHL treatment.^3^

Future research should focus on identifying biomarkers capable of selecting patients who may benefit from targeted biological therapies and improving strategies to maximize cochlear drug delivery.

### Conclusion

Current evidence regarding biological agents for the treatment of sudden sensorineural hearing loss remains limited and inconclusive.

Although preclinical studies and early-phase clinical trials provide biological plausibility and promising signals of efficacy, robust randomized controlled trials demonstrating consistent clinical benefit are lacking.^262^, ^263^, ^264^, ^265^, ^266^, ^267^, ^268^, ^269^, ^270^, ^271^, ^272^, ^273^, ^274^, ^275^, ^276^, ^277^

At present, there is insufficient high-quality evidence to recommend routine clinical use of: Anti-TNF agents; Mycophenolate mofetil; Methotrexate; Neurotrophic factors; Mesenchymal stem cells; AM-111; outside research protocols.

Corticosteroids remain the established therapeutic approach for SSNHL.^3^

Biological therapies should be reserved for carefully designed clinical trials until further evidence clarifies their safety, efficacy, and appropriate indications.

Ongoing research is essential to identify specific patient subgroups who may benefit from targeted biologic interventions and to optimize therapeutic protocols.

## Statement 14 ‒ Auditory rehabilitation in sudden sensorineural hearing loss

Clinicians should counsel patients with persistent hearing loss and/or tinnitus following SSNHL regarding available auditory rehabilitation options, including hearing aids, CROS systems, bone conduction devices, and cochlear implantation, according to individual hearing status, functional needs, and patient preferences. Strong recommendation.

### Action statement

Quality improvement opportunity: Improve functional hearing outcomes, communication abilities, tinnitus burden, social participation, and quality of life in patients with persistent auditory deficits following SSNHL through timely referral and individualized auditory rehabilitation.

Aggregate evidence quality: Grade B, based on systematic reviews, randomized controlled trials, prospective cohort studies, and observational studies demonstrating meaningful benefits of auditory rehabilitation technologies in patients with residual hearing loss after SSNHL.

Level of confidence in the evidence: High.

Benefits: Improved speech perception; enhanced communication in quiet and noisy environments; improved sound awareness; improved sound localization in selected patients; reduction in tinnitus burden; improved quality of life; improved social participation; reduced hearing handicap; restoration of binaural hearing cues in cochlear implant recipients; long-term functional rehabilitation.

Risks, harms, costs: Financial costs of rehabilitation devices; need for ongoing device maintenance and follow-up; surgical risks associated with cochlear implantation and bone conduction devices; variable adaptation periods; potential dissatisfaction if expectations are unrealistic; limited benefit of some devices in specific listening situations.

Benefits-harm assessment: Preponderance of benefit over harm.

Value judgments: The guideline panel placed a high value on restoration of hearing function and quality of life in patients with persistent hearing loss after SSNHL. The panel recognized that no single rehabilitation strategy is appropriate for all patients and that treatment selection should be individualized according to audiometric characteristics, hearing goals, tinnitus severity, contralateral hearing status, and patient expectations.

Intentional vagueness: The statement does not establish rigid audiometric thresholds for selecting hearing aids, CROS systems, bone conduction devices, or cochlear implants. Device selection should be individualized based on functional performance, residual hearing, patient-specific factors, and local availability of rehabilitation services.

Role of patient preferences: High. Selection of auditory rehabilitation modality should be based on shared decision-making, taking into account patient goals, lifestyle, occupational demands, cosmetic concerns, willingness to undergo surgery, tinnitus burden, expected benefits, and financial considerations.

Exceptions: Patients who achieve complete hearing recovery, patients who decline rehabilitation, patients medically unfit for surgical interventions, or patients unable to participate in rehabilitation programs.

Policy level: Strong recommendation.

Differences of opinion: Minor disagreement existed regarding the optimal timing of cochlear implantation and specific candidacy criteria for patients with single-sided deafness and asymmetric hearing loss. However, there was broad agreement that all patients with persistent hearing deficits after SSNHL should be counseled regarding available rehabilitation options and referred for appropriate auditory rehabilitation when indicated.

**Supporting text**

## Introduction

In adults, the most common cause of acquired SSD (single sided deafness) is idiopathic, or SSNHL (sudden sensorineural hearing loss), with other potential causes including cholesteatoma and infections, cerebellopontine angle tumors and, more rarely, head trauma, autoimmune diseases, and Ménière's disease.^278^

The prevalence of unilateral hearing loss is estimated at 7.2%.^279^

Kay-Rivest et al. estimated that the prevalence of SSD in the United States is around 0.11%–0.14% (271,122 to 345,064 adults).^280^

Approximately 93% of individuals with SSD believe that their hearing loss has a negative effect on social interactions.^281^

SSD is often associated with various symptoms that can significantly impair a person's daily life.

Tinnitus is a common problem. Between 54% and 84% of adults with SSD present with very bothersome tinnitus.^281^^,^^282^

Patients may also present with hyperacusis,^283^ aural fullness, and vestibular system alterations, mainly in cases of cochleo-vestibular involvement such as in Ménière's disease.^284^^,^^285^

In individuals with normal hearing, unilateral auditory stimulation causes predominantly contralateral auditory cortex activation (contralateral dominance).^286^^,^^287^

Unilateral hearing loss with disruption of binaural inputs results in cerebral reorganization with weakening in the representation of the deprived ear and strengthening in the representation of the intact ear (“auditory preference syndrome”.^288^^,^^289^

Cerebral reorganization is detectable five weeks after the onset of SSD in adults.^290^

SSD has been associated with increased anxiety levels, communication difficulty in the presence of background noise, and decreased self-esteem.^291^^,^^292^

Communication difficulty in noisy environments leads patients to withdraw from social situations, impacting personal and professional relationships.

The impact also extends to general well-being. People with unilateral hearing loss are more likely to report health problems, dissatisfaction, and loneliness than those with normal hearing.^293^^,^^294^

### Rehabilitation options after SSNHL

The choice between Hearing Aids (HA), Cochlear Implants (CI), or Bone Anchored Hearing System (BAHS) for SSNHL rehabilitation depends on multiple factors, including: Degree of hearing loss; Response to initial treatment; Presence of tinnitus and vertigo; Patient expectations; Surgical eligibility.

After clinical treatment of SSNHL, if hearing recovery is partial or absent, amplification is recommended.

Conventional hearing aids are the first line of amplification for mild to moderate hearing losses.

However, for severe to profound losses, or when hearing aids do not provide adequate benefit, cochlear implants emerge as a superior option for speech perception and other functional improvements.^295^

The evolution of cochlear implant criteria, especially approval for unilateral deafness and asymmetric hearing loss, has transformed the treatment landscape for SSD.^118^^,^^119^

Previously, these patients were treated primarily with Contralateral Routing of Signal/Bilateral Contralateral Routing of Signal (CROS/BiCROS) hearing aids or bone conduction devices.

Currently, cochlear implants offer significant auditory benefits, tinnitus suppression, and quality of life improvement that surpass traditional options for many patients.^119^

Healthcare service users express uncertainty about choosing treatment options for SSD, often due to lack of clarity about their benefit.^296^

Kitterick et al. evaluated in a systematic review studies on the effectiveness of different interventions (CROS, BAHS, and CI) in SSD and identified that results have been somewhat biased toward evaluating functional impairments for which measures are available and widely used, such as speech perception tests in noise.^295^

The difficulties imposed by SSD may also affect the individual's psychological and social well-being, and therefore outcomes assessing the impact on health and general well-being are also relevant and potentially important.^297^^,^^298^

### Conventional hearing aids

For patients with residual hearing after sudden sensorineural hearing loss, conventional hearing aids remain an important rehabilitation option.

The benefit depends on: Degree of residual hearing; Speech discrimination ability; Dynamic range; Patient adaptation.

When usable hearing remains in the affected ear, amplification may improve speech perception and communication ability.^299^^,^^300^

However, in cases progressing to Single-Sided Deafness (SSD), conventional amplification usually provides limited benefit because the affected ear cannot effectively process acoustic information.^295^^,^^301^

### Contralateral Routing of Signal (CROS) hearing aids

Contralateral Routing of Signal (CROS) systems were developed to overcome the head shadow effect observed in SSD.^303^

These devices capture sound from the deaf side and transmit it wirelessly to the normal-hearing ear.

The main advantage is improved awareness of sounds originating from the affected side.^304^

However, CROS devices do not restore binaural hearing because auditory input continues to be processed by only one cochlea.

Therefore, limitations remain regarding: Sound localization; Spatial hearing; Speech perception in complex noisy environments.^295^^,^^304^

Despite these limitations, CROS systems may represent a non-surgical rehabilitation option, particularly for patients who: Are not candidates for cochlear implantation; Prefer non-invasive approaches; Have contraindications for surgery.^304^

### Bone Anchored Hearing Systems (BAHS)

Bone Anchored Hearing Systems (BAHS) are another rehabilitation strategy for SSD.

They transmit sound from the impaired side to the contralateral functioning cochlea through bone conduction.^305^

Similar to CROS devices, BAHS improves access to sounds arriving from the deaf side and reduces the head shadow effect.^305^^,^^306^

However, because stimulation occurs through the normal-hearing cochlea, BAHS also does not restore true binaural processing.^295^

Studies have demonstrated improvement in: Speech perception when speech originates from the deaf side; Hearing-related quality of life; Patient satisfaction.^305^, ^306^, ^307^

Nevertheless, improvements in sound localization are limited, since interaural time differences and interaural level differences cannot be restored.^308^

### Comparison between CROS and BAHS

Both CROS and BAHS act primarily as rerouting strategies.

Their benefits include: Reduction of head shadow effect; Improved sound awareness from the affected side; Better communication in selected listening situations.^295^^,^^304^^,^^305^

Their limitations include: Lack of binaural restoration; Limited improvement in localization; Persistent difficulty understanding speech in complex noise environments.^295^^,^^308^

Therefore, although CROS and BAHS remain useful options, cochlear implantation has increasingly become the preferred rehabilitation strategy for selected patients with SSD because it is the only modality capable of restoring bilateral auditory input.^119^

### Cochlear implantation in SSD after SSNHL

Cochlear Implantation (CI) has become an increasingly established treatment option for patients with Single-Sided Deafness (SSD), including cases resulting from sudden sensorineural hearing loss.^119^

Unlike CROS and BAHS systems, cochlear implants stimulate the auditory pathway of the affected ear, restoring bilateral auditory input and allowing partial recovery of binaural hearing mechanisms.^309^, ^310^, ^311^, ^312^, ^313^

Duration of deafness has historically been considered an important prognostic factor for cochlear implant outcomes because prolonged auditory deprivation may lead to cortical reorganization.^309^

However, recent evidence suggests that even patients with long-standing SSD may benefit from cochlear implantation, although earlier implantation may optimize outcomes.^310^^,^^311^

Systematic reviews and meta-analyses have demonstrated that cochlear implantation in SSD provides significant improvement in: Speech perception in noise; Sound localization; Tinnitus reduction; Hearing-related quality of life.^312^, ^313^, ^314^, ^315^

### Restoration of binaural hearing

Binaural hearing depends on the integration of auditory information from both ears.

This allows: Head shadow effect reduction; Binaural summation; Binaural squelch; Sound localization using interaural timing and intensity differences.^308^^,^^319^

Patients with SSD lose access to these binaural cues, resulting in impaired localization and difficulty understanding speech in complex listening environments.^308^

Cochlear implantation is currently the only rehabilitation strategy capable of restoring auditory stimulation to the deaf ear and providing bilateral auditory input.^119^^,^^318^

### Sound localization after cochlear implantation

Several studies have demonstrated improvement in sound localization after cochlear implantation for SSD.^317^^,^^319^, ^320^, ^321^, ^322^

Although performance does not usually return completely to normal binaural hearing levels, CI users demonstrate significant improvement compared with unaided conditions or rerouting technologies such as CROS and BAHS.^320^, ^321^, ^322^

Arndt et al. demonstrated that cochlear implantation provided superior outcomes compared with CROS and BAHS regarding localization ability and speech perception in complex listening situations.^322^

### Comparative effectiveness: CI versus CROS versus BAHS

The CINGLE randomized controlled trial compared cochlear implantation, CROS, BAHS, and no treatment in adults with SSD.^324^

The study demonstrated that cochlear implantation provided the greatest overall benefit, including improvement in: Speech perception in noise; Sound localization; Tinnitus burden; Disease-specific quality of life.^324^

These findings support the role of CI as a preferred rehabilitation option in appropriately selected SSD patients.^119^^,^^318^^,^^324^

### Cortical plasticity after SSD and cochlear implantation

Unilateral auditory deprivation results in central auditory pathway reorganization, with increased dominance of the normal-hearing ear.^288^, ^289^, ^290^

Cochlear implantation may partially reverse maladaptive cortical plasticity by restoring stimulation to the deprived auditory pathway.^316^

Neuroimaging studies suggest that auditory cortical reorganization may occur after CI use, supporting improved binaural processing and functional outcomes.^316^

The potential reversibility of cortical changes reinforces the importance of considering auditory rehabilitation rather than long-term observation alone after irreversible SSNHL.^309^^,^^316^

### Tinnitus outcomes after rehabilitation

Tinnitus is one of the most frequent and disabling symptoms associated with single-sided deafness following sudden sensorineural hearing loss.^281^^,^^282^

The prevalence of bothersome tinnitus in SSD patients is high and may significantly affect: Sleep; Concentration; Emotional well-being; Quality of life.^281^^,^^282^

Conventional hearing aids may provide tinnitus relief in selected patients with residual hearing by increasing auditory stimulation and reducing the contrast between tinnitus perception and environmental sound.^302^

However, their effectiveness is limited in profound hearing loss because acoustic stimulation of the affected ear is not possible.^302^

CROS and BAHS devices may improve communication difficulties associated with SSD but generally have limited impact on tinnitus because they do not restore stimulation of the deprived auditory pathway.^304^^,^^305^

Cochlear implantation has demonstrated significant tinnitus suppression in SSD patients.^314^^,^^315^^,^^325^^,^^326^

Several systematic reviews and meta-analyses report clinically meaningful reductions in tinnitus severity scores after cochlear implantation.^314^^,^^315^

The proposed mechanisms include: Restoration of auditory input; Reorganization of maladaptive cortical activity; Reduction of abnormal spontaneous neural activity associated with auditory deprivation.^316^

Therefore, in patients with SSD and severe tinnitus after SSNHL, cochlear implantation may provide dual benefits: Improvement of hearing function; Reduction of tinnitus burden.^119^^,^^315^

### Cost-effectiveness considerations

Although cochlear implantation requires higher initial investment compared with CROS or BAHS, long-term analyses suggest favorable cost-effectiveness in selected SSD populations.^327^, ^328^, ^329^, ^330^

Economic evaluations demonstrate that benefits related to: Improved communication; Increased Quality-Qdjusted Life Years (QALYs); Reduction of tinnitus-related disability; May justify the cost of implantation over time.^327^, ^328^, ^329^, ^330^

Seebacher et al. demonstrated that cochlear implantation for SSD can represent a cost-effective intervention when long-term auditory and quality-of-life benefits are considered.^327^

Similar findings have been reported in European health economic analyses evaluating unilateral deafness rehabilitation.^328^, ^329^, ^330^

### Clinical recommendation

Patients with persistent hearing impairment after sudden sensorineural hearing loss should undergo individualized auditory rehabilitation assessment.

For mild-to-moderate residual hearing loss: Conventional hearing aids should be considered as first-line rehabilitation.

For severe-to-profound unilateral hearing loss or SSD: CROS and BAHS may improve awareness of sound from the affected side but do not restore binaural hearing.^295^^,^^304^^,^^305^ Cochlear implantation should be considered in appropriate candidates because it provides superior restoration of bilateral auditory input, improves speech perception in noise, enhances sound localization, reduces tinnitus, and improves quality of life.^119^^,^^312^, ^313^, ^314^, ^315^, ^316^, ^317^, ^318^, ^319^, ^320^, ^321^, ^322^, ^323^, ^324^

### Conclusion

Sudden sensorineural hearing loss may result in persistent auditory impairment requiring long-term rehabilitation.

Although CROS and BAHS remain valid options for selected patients, they function primarily as rerouting strategies and do not restore true binaural hearing.^295^, ^296^, ^297^, ^298^, ^299^, ^300^, ^301^, ^302^, ^303^, ^304^, ^305^

Cochlear implantation represents the only currently available rehabilitation strategy capable of stimulating the affected auditory pathway and partially restoring binaural auditory processing.^119^^,^^318^

Current evidence supports cochlear implantation as an effective treatment for SSD after SSNHL, with benefits in: Speech perception; Sound localization; Tinnitus suppression; Hearing-related quality of life.^312^, ^313^, ^314^, ^315^, ^316^, ^317^, ^318^, ^319^, ^320^, ^321^, ^322^, ^323^, ^324^, ^325^, ^326^

Future research should focus on defining optimal timing after SSNHL, identifying predictors of benefit, and improving access to rehabilitation strategies.

## Statement 15 ‒ Routine post-treatment follow-up

Clinicians should obtain follow-up audiometric evaluation at the completion of treatment, within 2- to 3-months, and within 6-months after SSNHL onset to assess hearing recovery, identify delayed improvement, and guide auditory rehabilitation. Additional follow-up through 12-months and annual monitoring thereafter may be considered in patients with persistent hearing loss, hearing instability, significant tinnitus, or ongoing rehabilitation needs. Strong recommendation.

### Action statement

Quality improvement opportunity: Standardize post-treatment follow-up in patients with SSNHL, improve detection of delayed hearing recovery, facilitate timely initiation of salvage therapy and auditory rehabilitation, and optimize long-term hearing outcomes and quality of life.

Aggregate evidence quality: Grade B, based on systematic reviews, meta-analyses, randomized controlled trials, observational studies, and consistent expert consensus supporting structured audiometric monitoring after treatment for SSNHL.

Level of confidence in the evidence: High.

Benefits: Document hearing recovery; identify delayed or progressive improvement; detect hearing instability or recurrence; guide decisions regarding salvage therapy; facilitate timely auditory rehabilitation; improve patient counseling; monitor long-term outcomes; support management of tinnitus and associated symptoms; optimize hearing-related quality of life.

Risks, harms, costs: Costs associated with repeated audiometric evaluations; potential inconvenience related to follow-up visits; minimal additional healthcare resource utilization. No significant clinical harms are associated with audiometric monitoring.

Benefits-harm assessment: Preponderance of benefit over harm.

Value judgments: The guideline panel placed a high value on longitudinal assessment of hearing recovery because a substantial proportion of patients may continue to improve after completion of initial therapy. Structured follow-up also facilitates identification of patients who may benefit from additional interventions, including salvage treatment, hearing rehabilitation, tinnitus management, or cochlear implantation.

Intentional vagueness: The statement does not mandate a rigid follow-up schedule for all patients. Although audiometric evaluation at treatment completion and within 6 months is recommended, additional assessments may be individualized according to hearing recovery, symptom persistence, hearing instability, rehabilitation needs, and clinician judgment.

Role of patient preferences: Limited. Patients may participate in decisions regarding the frequency and duration of long-term monitoring; however, follow-up audiometric assessment is considered an essential component of appropriate SSNHL management.

Exceptions: Patients with complete and stable recovery who decline further monitoring, patients unable to attend follow-up evaluations, or patients with severe medical conditions limiting audiologic reassessment.

Policy level: Strong recommendation.

Differences of opinion: Minor disagreement existed regarding the optimal duration and frequency of long-term audiometric follow-up beyond six months. However, there was broad consensus that audiometric evaluation should be performed at treatment completion and during the first six months after SSNHL onset, with additional follow-up individualized according to clinical circumstances.

**Supporting text**

Post-treatment follow-up for sudden hearing loss should be established to document hearing recovery, identify recurrence or fluctuation, and plan rehabilitation for residual hearing loss and/or tinnitus.^3^^,^^153^^,^^331^

Pure-tone audiometry is the most accurate, cost-effective, and widely used method in the literature for monitoring the effectiveness of medical interventions in sudden sensorineural hearing loss (SSNHL).

Because most patients do not have pre-event audiometric data, after confirmation that hearing was subjectively symmetrical prior to the event, it is reasonable to use the contralateral, unaffected ear as a baseline reference.

Unless pre-existing hearing asymmetry is known or suspected, the non-affected ear should be considered the standard for comparison when assessing hearing recovery.^3^

Early post-treatment audiometry, performed at the end of therapy, allows evaluation of the benefit of the initial intervention and helps guide decisions regarding salvage therapy.^3^^,^^154^, ^155^, ^156^

Patients with incomplete hearing recovery should undergo reevaluation within 2–3 months after completion of treatment, even when salvage therapy is not administered, as delayed hearing recovery may occur, particularly in cases with partial initial improvement.

In a retrospective study including 156 patients, 55 (45.5%) demonstrated delayed hearing recovery between 3- and 12-weeks following oral corticosteroid therapy.

Among these patients, 78.2% experienced recovery within the first month, 5.5% between 1- and 2-months, 12.7% between 2- and 3-months, and 3.6% after 3-months.^332^

Additional studies have reported hearing recovery at 3-months, while others have noted hearing stabilization occurring between 2- and 4-months, 2- and 6-months, or as late as 12-months.^3^

In 2020, a systematic review and meta-analysis of randomized controlled trials compared systemic and intratympanic corticosteroids as first-line treatments for SSNHL.

Follow-up periods for outcome assessment ranged from 21- to 90-days across the seven included studies.^331^

In 2022, another systematic review and meta-analysis evaluated three prospective randomized studies involving a total of 88 participants who received hyperbaric oxygen therapy in the intervention group and 62 participants who received medical therapy alone in the control group.^153^

Among the included studies, one did not specify the timing of post-treatment audiometric evaluations, while the others performed audiometry at 1- and 3-months after treatment.^153^

In 2024, a multicenter, randomized, double-blind study compared oral doses of SENS-401 with placebo; all patients also received corticosteroids.

Audiometric assessments were conducted on days-7, -14, and -28 (end of the study treatment period) and on day-84, corresponding to 8-weeks after treatment completion.^333^

These findings highlight the considerable variability in the literature regarding the duration of long-term audiometric follow-up after treatment for SSNHL.

Nevertheless, there is consensus that follow-up audiometry at 6-months after the onset of sudden hearing loss should be performed regardless of the degree of initial hearing recovery.^3^

Follow-up may be extended to 12-months or longer in cases of hearing instability or fluctuation, or when auditory rehabilitation with hearing aids or cochlear implantation is indicated.

Early discussion of auditory rehabilitation options, vestibular rehabilitation, and tinnitus management is recommended when residual symptoms persist.

Verification of the proper functioning of hearing amplification devices should be performed annually or as clinically indicated.^156^^,^^331^

In addition, management of or referral for associated comorbidities is advisable, as metabolic syndrome has been identified as an unfavorable prognostic factor in SSNHL.^332^

Despite this association, the precise mechanisms driving the recovery process remain unclear.

It has been postulated that cellular defense systems and intrinsic repair strategies, notably synaptogenesis and synaptic regeneration, may play a substantial role in auditory recovery.^333^

The overall objective of post-treatment follow-up is to provide comprehensive patient care, facilitate auditory rehabilitation, and identify potential etiologies that may become evident at later stages following the episode of sudden hearing loss.^3^

### Quality of evidence

Grade B: based on systematic reviews and meta-analyses of randomized clinical trials and observational studies showing a preponderance of benefits compared to harms.

### Differences of opinion

The authors agree that an audiological examination should be performed: At the end of the initial therapy; After 2–3 months of the initial therapy or at the end of salvage therapy, if applicable; After 6-months of treatment.

However, there is some disagreement regarding when long-term follow-up audiometry should be obtained.

### Recommendations

• Early audiometry: should be performed during and immediately after the end of treatment or after salvage therapy, if performed.
• Audiometry at 2–3 months: should be indicated due to the possibility of progressive hearing improvement in this interval.
• Audiometry at 6-months: should be indicated to document hearing outcome, identify late recovery, and plan hearing rehabilitation.
• Semiannual audiometry up to 12-months: recommended in cases with hearing instability or persistent unilateral hearing loss.
• Annual monitoring: recommended to verify hearing stability and the need for hearing rehabilitation with hearing aids or cochlear implants in patients without improvement after more than 3-months of hearing aid use.

Unilateral cochlear implantation may be considered as a treatment option when hearing loss is severe-to-profound and accompanied by significant tinnitus, with no improvement after more than 3-months of hearing aid use.

Recommendation based on systematic reviews and meta-analyses with a preponderance of benefits compared to harms.

## ORCID ID

Rogério Hamerschmidt: 0000-0002-7722-6409

Robinson Koji Tsuji: 0000-0001-5164-0786

Arthur Menino Castilho: 0000-0002-9024-8004

André Luiz Lopes Sampaio: 0000-0001-7611-1303

Denis de Melo Pinto Rangel: 0000-0002-6437-1535

Eduardo Tanaka Massuda: 0000-0003-3441-5709

Fabio de Alencar Rodrigues Junior: 0009-0007-9393-9664

Felippe Felix: 0000-0001-5314-6546

Giulliano Luchi: 0009-0003-2497-1848

Gustavo Lara Rezende: 0000-0003-2652-9570

Henrique Furlan Pauna: 0000-0002-5575-7030

Joel Lavinsky: 0000-0002-0337-5704

Juliana Gusmão de Araujo: 0000-0001-7853-3016

Kiara Kalline Medeiros: 0000-0001-5540-5513

Lucas Moura Viana: 0000-0001-5872-4915

Márcio Cavalcante Salmito: 0000-0001-6316-3358

Mariana de Carvalho Leal: 0000-0003-3358-2791

Marina Bernardes: 0000-0002-9051-0180

Matheus Pedrosa Tavares: 0000-0002-4288-740X

Miguel Angelo Hipolyto: 0000-0001-9688-782X

Natália Carasek Matos Cascudo: 0000-0002-0016-9020

Natália Quinhone Shigematsu: 0009-0008-7634-9141

Norma de Oliveira Penido: 0000-0003-3496-410X

Pauliana Lamounier: 0000-0003-3552-5264

Rafael da Costa Monsanto: 0000-0002-9124-593X

Luiz Rodolpho Penna Lima Júnior: 0009-0008-7004-8006

Thaís Gomes Abrahão Elias: 0000-0001-6593-4785

Vagner Antonio Rodrigues Silva: 0000-0002-7335-4489

## Authorship statement

All authors meet the authorship criteria, reviewed and approved the final version of the manuscript, and agree to be accountable for all aspects of the work.

## Ethical approval

Not applicable.

## Declaration of Generative AI and AI-assisted technologies in the writing process

None.

## Funding

None.

## Data availability statement

Not applicable.

## Declaration of competing interest

The authors declare no conflicts of interest.
